# Sex-specific efficacy of curcumin-loaded nanoparticles and insulin for ocular-cardiac complications in type 2 diabetes mice

**DOI:** 10.1016/j.isci.2026.116594

**Published:** 2026-07-02

**Authors:** Swetha R. Allamreddy, Raghu Ganugula, Meenakshi Arora, Sabrina Ingram, Subhash Dwivedi, Richard Friend, Rita Basu, M.N.V. Ravi Kumar

**Affiliations:** 1 The Center for Convergent Bioscience and Medicine (CCBM), The University of Alabama, Tuscaloosa, AL 35401, USA; 2 Department of Translational Science and Medicine, College of Community Health Sciences, The University of Alabama, Tuscaloosa, AL 35401, USA; 3 Alabama Life Research Institute, The University of Alabama, Tuscaloosa, AL 35401, USA; 4 Department of Biological Sciences, The University of Alabama, Tuscaloosa, AL 35401, USA; 5 Center for Free Radical Biology, University of Alabama at Birmingham, Birmingham, AL 35294, USA; 6 Nephrology Research and Training Center, Division of Nephrology, Department of Medicine, University of Alabama at Birmingham, Birmingham, AL 35294, USA; 7 Family, Internal, and Rural Medicine, College of Community Health Sciences, The University of Alabama, Tuscaloosa, AL 35401, USA; 8 Division of Endocrinology, Diabetes, and Metabolism, School of Medicine, Marnix E. Heersink School of Medicine, The University of Alabama, Birmingham, AL 35294, USA

**Keywords:** diabetic complications, curcumin nanoparticles, eye-heart axis, inflammation, lymphangiogenesis, pressure correlation, sex-differences

## Abstract

Current Type 2 diabetes therapies inadequately address multi-organ complications. We evaluated bivalent nanoparticles encapsulating curcumin, combined with insulin, against diabetic complications. Our data indicate shared pathophysiological mechanisms driving the association between retinal and cardiac injury, specifically systemic inflammation and mitochondrial dysregulation across both organs. Retinal stress (HIF1α, REDD1, VEGF) preceded cardiac remodeling, characterized by lymphangiogenesis (LYVE1, VEGFR3) and impaired PPARα/ PGC1α signaling. Furthermore, we identified a strong pathological correlation between intraocular and systemic blood pressures as an *in-vivo* physiological readout of widespread vascular dysregulation. While males exhibited an earlier onset, females progressed more rapidly to complications. The combination therapy proved highly effective in both sexes. While females exhibited a more pronounced reduction in systemic inflammation and pathological pressure correlation, males demonstrated robust metabolic and structural preservation. These findings highlight ocular hemodynamics as an early physiological indicator of cardiac complications, supporting this sex-specific nanotherapeutic strategy, combined with insulin, to mitigate diabetic complications.

## Highlights

• Retinal stress markers show a concomitant association with early cardiac remodeling
• Vascular and mitochondrial changes mark parallel ocular and cardiac progression
• IOP and MAP correlation serve as physiological indicators of vascular dysregulation
• Bivalent curcumin nanotherapy with insulin restores hemodynamic homeostasis

## Graphical abstract

**Figure:**
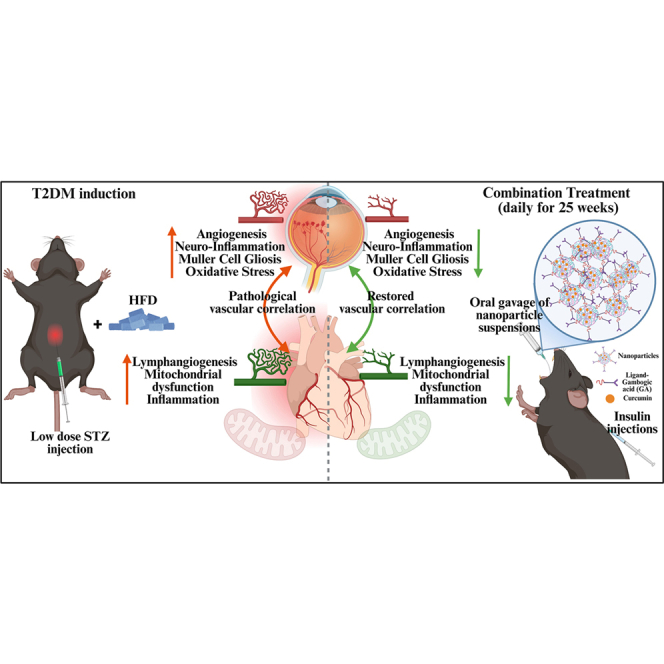

## Teaser

Health sciences; Therapeutics; Nanotechnology; Medical biotechnology

## Introduction

Diabetes mellitus (DM) is a global health problem, with projections estimating 853 million cases by 2050, 90% of which are type 2 diabetes mellitus (T2DM).^1^ This metabolic disorder affects multiple organ systems through complex pathways involving polyol pathway activation, increased advanced glycation end products, protein kinase C activation, oxidative stress, and chronic inflammation. The crosstalk among these pathways drives the progression of multi-organ complications.^2^^,^^3^

Diabetic cataract and retinopathy (DR) are the primary causes of vision loss associated with diabetes. While glycemic control delays cataractogenesis, surgical extraction of cataract remains the only definitive treatment, but is often complicated by delayed healing and persistent inflammation in patients with diabetes.^4^ DR presents a more complex pathology, marked by neurovascular destabilization, and degenerative changes in the retina’s blood-retinal barrier (BRB).^5^^,^^6^^,^^7^ In DR, under sustained hyperglycemia and inflammation, a vicious cycle of endothelial damage, pericyte loss, and vascular leakage disrupts the neurovascular unit (NVU).^5^^,^^6^ Current monotherapies targeting only inflammatory or angiogenic factors are often insufficient,^6^ necessitating a need for a multi-targeted therapeutic approach that protects vascular and neural components while reducing systemic inflammation and achieving glucose homeostasis.

Parallel to DR, the diabetic heart is equally vulnerable to hyperglycemia-induced injury. Diabetic cardiomyopathy (DCM) develops through shared mechanisms, including impaired insulin (INS) signaling, mitochondrial dysfunction, and chronic low-grade inflammation.^8^ Morbidity and mortality rates are high in patients with DCM, with females showing higher susceptibility.^9^ The current management of DCM, in addition to anti-hyperglycemic agents, includes targeted therapeutic agents such as statins, renin blockers, and β-receptor blockers. However, these often treat symptoms in isolation rather than addressing DCM as a systemic metabolic sequela.^10^ Consequently, there is an urgent need for interventions that simultaneously target the metabolic and inflammatory drivers of both ocular and cardiac complications.

Polyphenols are widely studied for managing diabetic complications due to their ability to modulate multiple signaling pathways, but their use is limited by poor solubility and permeability.^11^^,^^12^ Studies have shown that receptor-targeted delivery using a ligand improves bioavailability and drug efficacy with minimal side effects and dosage requirements.^13^ Building on this, our lab developed a receptor mediated bivalent nanoparticles (NPs) system (nGA_2_-CUR), wherein two molecules of gambogic acid (GA) are linked to a biodegradable polymer (PLGA).^14^ GA acts as a ligand that binds non-competitively to transferrin receptors expressed on intestinal cells,^15^ improving the oral bioavailability of curcumin (CUR) as a model drug.^14^ Given the extensive evidence supporting CUR’s efficacy in the management of diabetes and metabolic conditions, CUR is an ideal candidate for encapsulation into these NPs in this study. Furthermore, our previous studies with these CUR-loaded NPs (CUR-NPs) showed promising results in type 1 diabetic rat models when combined with subcutaneous INS injections (INSs.c.).^16^^,^^17^^,^^18^

For this study, we transitioned to a more clinically representative T2DM model, induced by a high-fat diet (HFD) and low-dose streptozotocin (STZ).^19^^,^^20^ This model accurately reflects the progression of human T2DM, including obesity-induced INS resistance, compensatory β-cell dysfunction, and the eventual development of ocular and cardiac complications, offering high translational value.^21^^,^^22^^,^^23^ Another key aspect of this study is the inclusion of both sexes to address biological sex as a variable. Rather than treating sex as a confounding factor, our experimental design was explicitly powered to capture distinct, sex-dimorphic disease progression patterns and evaluate whether the therapeutic efficacy of our nanotherapeutic strategy manifests differently in male versus female cohorts. This allowed us to capture distinct disease progression patterns, where males exhibit earlier onset, yet females demonstrate a more rapid progression to severe complications.^24^

Here, we present a combination therapy utilizing highly bioavailable CUR-NPs alongside basal INS support. Beyond stabilizing glucose levels and reducing systemic inflammation, this regimen significantly delayed the progression of cataracts and diabetic retinopathy. Given the shared vascular susceptibility of the retina and heart, we hypothesized that retinal vascular stress serves as an early indicator of cardiovascular disease (CVD).^25^ We provide *in-vivo* physiological evidence of hemodynamic association, demonstrated by a strong pathological correlation between intraocular pressure (IOP) and mean arterial pressure. Our data suggest that inflammation, metabolic disturbances, and endothelial dysfunction drive concomitant pathology in both organs,^26^ identifying ocular hemodynamics as an early indicator of parallel heart complications and offering a critical window for intervention. Notably, our combination therapy effectively mitigated these early cardiac metabolic and endothelial changes. This study validates the potential of bioavailable anti-inflammatory therapy combined with INSs.c. for managing T2DM and underscores the importance of using clinically relevant models and including both sexes.

## Results

### Combination therapy mitigates hyperglycemia and reduces circulating inflammatory biomarkers in a sex-dependent T2DM model

**Figure 1:**
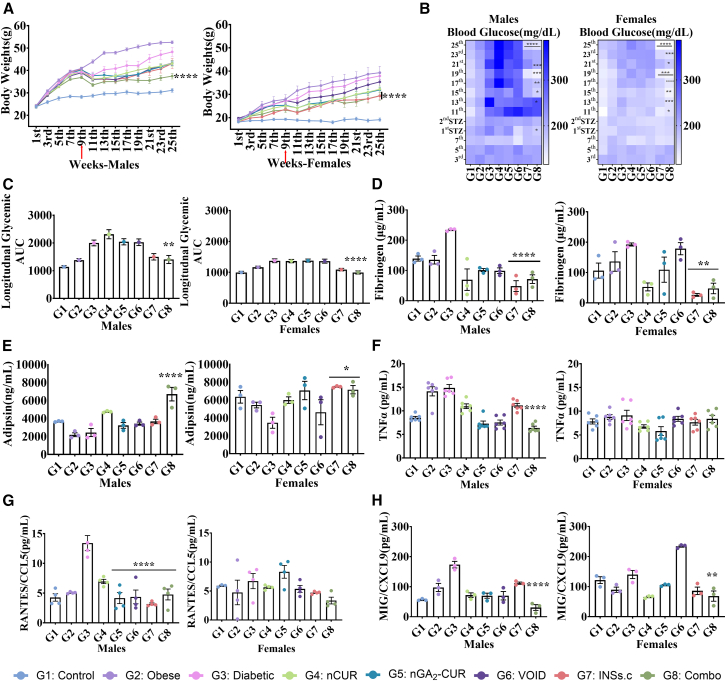
Combination therapy mitigates hyperglycemia and reduces circulating inflammatory biomarkers in a sex-dependent T2DM model (A and B) Line plots of the body weights collected throughout the study period. The red arrow indicates STZ injection at week 9 (*n* = 7–8 mice/ group) (B) Heat maps of blood glucose measured via tail prick over the 25-week study period (*n* = 7–8 mice/ group). (C) Cumulative glycemic burden (longitudinal area under the curve AUC) for blood glucose levels over a 16-week period of treatment (*n* = 7–8 mice/ group). (D and E) Plasma biomarkers of metabolic dysfunction, including D) Plasma Fibrinogen levels (*n* = 3 mice/ group) and E) Plasma Adipsin levels were measured (*n* = 3 mice/ group). (F–H) Inflammatory markers in Plasma were evaluated F) Plasma TNFα levels (*n* = 6 mice/group), (G) Plasma RANTES/ CCL5 levels (*n* = 3 mice/group), and H) MIG/ CXCL9 levels were measured (*n* = 3 mice/ group). Data are presented as mean ± SEM. Longitudinal body weights (A) and Heat maps (B) were assessed using a two-way ANOVA. All other datasets were analyzed using a one-way ANOVA followed by Tukey’s multiple comparison test. false discovery rate (FDR) correction was applied to the data sets, and all significant findings passed the FDR threshold (*q* < 0.05). While an all-versus-all comparison was performed, only the differences between the Diabetic group (G3) and the Combination group (G8) are annotated in the figure to highlight therapeutic efficacy against the disease model. Complete comparisons are provided in Table S3. The significance levels used in the analysis were as follows: ∗*p* < 0.05, ∗∗*p* < 0.01, ∗∗∗*p* < 0.001, and ∗∗∗∗*p* < 0.0001. See also Figures S3 and S4 and Table S3.

Mice on a HFD showed a steady increase in body weight, with males gaining more rapidly than females (Figure 1A). After STZ injections, a sharp drop in body weight marked the onset of T2DM, followed by a gradual increase, a trend more evident in males. Oral CUR NPs reduced weight gain, with the combination group (G8) showing the most normalization (Figure S3A).

Following STZ injections, males developed severe hyperglycemia within one week, while females took around two weeks (Figures 1B and S3B). Throughout the study, diabetic males experienced more significant glucose fluctuations than females. The combination therapy was consistently more effective in both sexes than INSs.c. therapy alone (Figure 1B). These results were supported by the area under the curve (AUC) analysis of the glucose line plots (Figure 1C), which shows the cumulative glycemic burden over the study duration. While INSs.c injections (G7 and G8) significantly reduced post-dosing glucose levels in both sexes (Figure S3C), there was no significant difference in the 24-h blood glucose profile (Figure S3D), highlighting the need for treatments that can maintain overnight glucose control. Plasma triglyceride levels were elevated across all diabetic groups, particularly males, but treatments had no significant effect (Figure S3E). Collectively, the body weight, glucose, and lipid profiles confirm that males are susceptible to a more rapid and aggressive T2DM onset compared to females.

To evaluate metabolic dysfunction, plasma fibrinogen and Adipsin were measured. Fibrinogen, an acute-phase protein and a CVD risk marker,^27^^,^^28^ was significantly reduced in both sexes across all treatment groups, with the greater reduction in G4, G7, and G8 (Figure 1D). Adipsin (complement factor D), a β-cell protective adipokine,^29^^,^^30^^,^^31^ was significantly improved in both sexes, with G8 showing the most significant effect. In males, improvement was also notable in G4, while females showed significant effects in G4, G5, and G7 (Figure 1E). Effect sizes were large for both markers, highlighting the significance of treatment (Figures S4A and S4B).

Chronic low-grade systemic inflammation drives T2DM progression and complications, making it a key target for management.^32^^,^^33^ Pro-inflammatory markers were evaluated in terminal plasma (Figures 1F–1H) due to their roles in INS resistance, endothelial dysfunction, and metabolic disorders.^34^^,^^35^ In males, TNFα and RANTES showed significant reductions in groups treated with nGA_2_-CUR (G5, G8), supported by large effect sizes (TNFα: ƞ^2^ = 0.84; RANTES: ƞ^2^ = 0.83). In females, both markers in diabetic mice did not differ significantly from healthy controls, reflected as small effect sizes (TNFα: ƞ^2^ = 0.30; RANTES: ƞ^2^ = 0.38). In contrast, MIG (CXCL9) decreased consistently across treatments in both sexes, with strong effect sizes (males: ƞ^2^ = 0.90; females: ƞ^2^ = 0.93), highlighting its robust responsiveness (Figures S4C–S4E).

Overall, diabetic males presented with a more aggressive systemic disease profile than females. Ranking these markers showed that combination therapy achieved the most favorable outcomes in both sexes (lowest sum and highest frequency of 1) (Figure S4F).

### Regulation of cellular stress markers and glial activation in the retinal neurovascular unit

Hyperglycemia induces hypoxia and oxidative stress, damaging capillary endothelial cells and triggering pathological angiogenesis, a hallmark of DR.^25^ Under hypoxic conditions, retinal Müller cells express the hypoxia marker Hypoxia-induced factor 1 α (HIF1α),^36^^,^^37^ and a redox sensor regulated in development and DNA damage-response 1 (REDD1),^38^^,^^39^ which we explored in our study.

**Figure 2:**
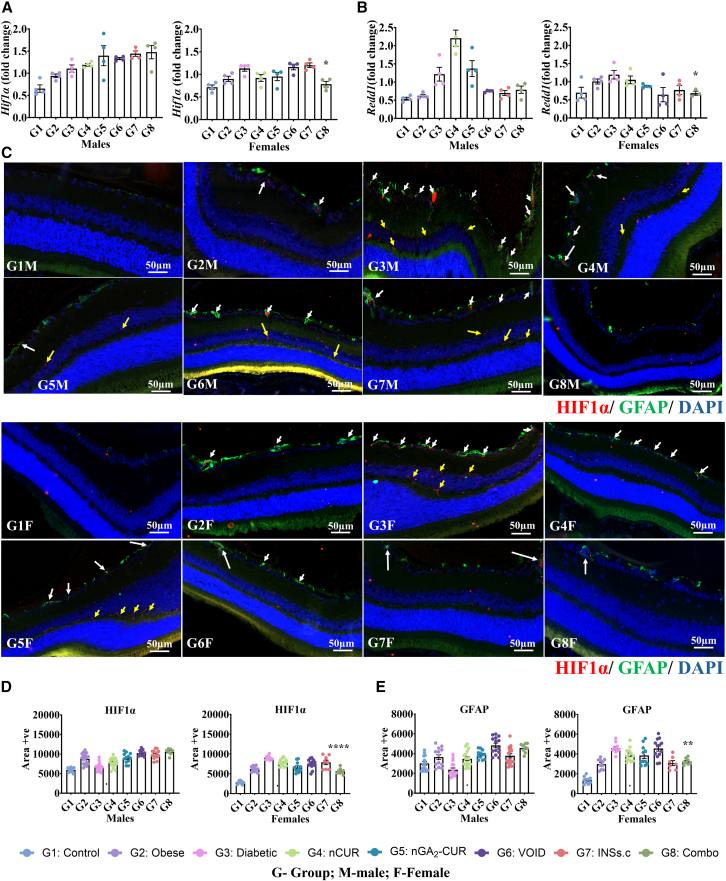
Regulation of cellular stress markers and glial activation in the retinal neurovascular unit (A and B) Real-time PCR analysis of mRNA levels for (A) *Hif1α* and (B) *Redd1*. Data are normalized to the housekeeping gene *β-actin* and presented as fold change relative to controls (*n* = 4 biological replicates/group; 4 technical replicates for each sample). (C) Representative immunofluorescence images of male and female retinal sections stained for HIF1α (red), GFAP (green), and DAPI (blue). White arrows indicate co-localization of HIF1α and GFAP in the retinal ganglion cell layer. Yellow arrows indicate the expression of HIF1α in the inner nuclear layer of retina. Images were acquired using a Zeiss Axioscan 7 microscope (20x magnification; scale bars, 50 μm). (D and E) Quantification of (D) HIF1α and (E) GFAP fluorescence intensity analyzed using ImageJ on defined retinal areas (75,000 μm^2^ per section) (*n* = 2–4 images from 4 animals/group). Data are presented as mean ± SEM. Unless otherwise noted, data were analyzed using a one-way ANOVA followed by Tukey’s multiple comparison test for all-versus-all comparisons. false discovery rate (FDR) correction was applied to the datasets, and all significant findings passed the FDR threshold (*q* < 0.05). In the female cohort, *Redd1* data did not follow a normal distribution (Shapiro-Wilk test). Consequently, statistical significance was determined using Welch’s *t* test (accounting for unequal variances). Comparison annotations shown in the figure highlight differences between the diabetic group (G3) and combination group (G8). Full statistical comparisons are provided in Table S4. The significance levels used in the analysis were as follows: ∗*p* < 0.05, ∗∗*p* < 0.01, ∗∗∗*p* < 0.001, and ∗∗∗∗*p* < 0.0001. See also Figure S5 and Table S4.

*Hif1α*, a cellular stress response marker, was upregulated in all diabetic groups (Figure 2A). Notably, male mice showed no response to treatment, whereas females receiving combination therapy (G8) had a significant reduction. *Redd1* showed upregulation in G3 groups with a significant decrease only in G8 females, whereas males showed a downregulation trend (Figure 2B). Effect size analysis supported these findings, showing strong to moderate treatment effects (*Hif1α*: ƞ^2^ = 0.67 males, 0.69 females; *Redd1*: ƞ^2^ = 0.83 males, 0.46 females), indicating that treatment produced biologically meaningful effects even when statistical significance was limited (Figures S5A and S5B). The absence of *Hif1α* and *Redd1* reduction in males suggests persistent stress signaling, while the response in females may be linked to enhanced sensitivity to therapy.

Immunostaining of retinal cross-sections for HIF1α (red) and glial fibrillary acidic protein (GFAP) (green) (Figure 2C) supported gene expression findings (Figures 2D and 2E). These markers were co-localized in the inner retina. GFAP, a marker for microglial activation, is upregulated in injured Müller cells and astrocytes in the retina. Similar to gene expression, no significant reductions in GFAP and HIF1α were observed in treated males. In contrast, G8 females showed notable reductions in both markers (Figures 2D and 2E). In females, HIF1α levels also decreased in the G5 group, while GFAP was significantly lowered with INSs.c. The effect size analysis indicated moderate to strong effects (HIF1α: ƞ^2^ = 0.67 males, 0.76 females; GFAP: ƞ^2^ = 0.49 males, 0.68 females), supporting the biological relevance of these changes, particularly in females (Figures S5C and S5D).

### Combination therapy attenuates angiogenic drivers and improves retinal fluid homeostasis markers

**Figure 3:**
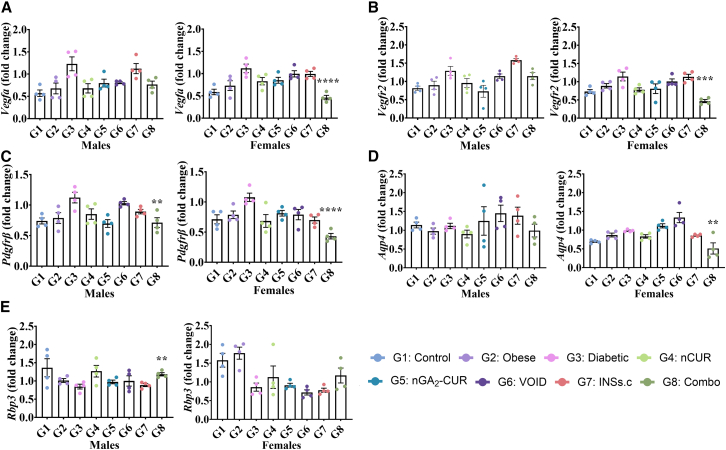
Combination therapy attenuates angiogenic drivers and improves retinal fluid homeostasis markers (A–C) Real-time PCR analysis of mRNA levels for key angiogenesis markers: (A) *Vegfa*, (B) *Vegfr2*, and (C) *Pdgfrβ* in male and female cohorts. (D and E) qRT-PCR analysis of relative (D) *Aqp4* and (E) *Rbp3* expression in the retina. Data are normalized to the housekeeping gene *β-actin* and presented as fold change relative to controls (*n* = 4 biological replicates/group; 4 technical replicates for each sample). Data are presented as mean ± SEM. Unless otherwise noted, data were analyzed using a one-way ANOVA followed by Tukey’s multiple comparison test for all-versus-all comparisons. False discovery rate (FDR) correction was applied to the dataset; all significant findings passed the FDR threshold (*q* < 0.05). In the male cohort, *Rbp3* data exhibited significant heterogeneity. Therefore, statistical significance was determined using a pre-planned pairwise Welch’s t-test (accounting for unequal variances), which identified a significant difference between the diabetic and control groups (∗∗*p* < 0.01). All comparisons were made relative to the diabetic group (G3), and only the comparisons between the diabetic group (G3) and the combination group (G8) are shown in Figure. Additional comparisons are summarized in Table S5. The significance levels used in the analysis were as follows: ∗*p* < 0.05, ∗∗*p* < 0.01, ∗∗∗*p* < 0.001, and ∗∗∗∗*p* < 0.0001. See also Figure S6 and Table S5.

In response to stress, gene expression of HIF1α-dependent angiogenic markers is upregulated in the retina, including Vascular endothelial growth factor A (*Vegfa*), its receptor *Vegfr2,* and platelet-derived growth factor receptor β (*Pdgfrβ*). They play a key role in promoting angiogenesis and maintaining vascular stability.^7^^,^^36^^,^^37^ Diabetic mice responded well to combination therapy, showing a significant reduction of the angiogenic markers, indicating anti-angiogenic potential (Figures 3A–3C). A similar but lesser effect was observed in the G4 and G5 groups. Effect size analysis further supported these findings, with *Vegfa* showing large effects (ƞ^2^ = 0.60 in males, 0.71 in females), *Vegfr2* (ƞ^2^ = 0.68 in males, 0.69 in females), and *Pdgfrβ* (ƞ^2^ = 0.60 in males, 0.65 in females), confirming the biological relevance of treatment-associated changes (Figures S6A–S6C).

Aquaporin 4 is primarily expressed by Müller cells, regulates water homeostasis, and its altered expression contributes to retinal edema.^5^ In our study, diabetic females exhibited significant upregulation of *Aqp4* levels, which was downregulated in G8, whereas diabetic males didn’t show any difference as compared to G1 (Figure 3D). It was further supported by effect size analysis with males showing minimal effects (ƞ^2^ = 0.23) but a strong effect in females (ƞ^2^ = 0.75) (Figure S6D).

Retinol-Binding Protein 3, a protective factor against DR, decreased levels are associated with the disease severity.^40^ In males, combination therapy led to significant *Rbp3* upregulation. In females, a similar trend was seen but was not statistically significant (Figure 3E). Effect size analysis indicated moderate-to-large effects (ƞ^2^ = 0.45 in males; 0.62 in females), highlighting treatment effectiveness despite statistical limitations (Figure S6E).

Rank-ordering the data, we observed varying levels of action among the intervention groups. Males showed the lowest scores in the groups receiving NPs (G4, G5, and G8). In contrast, females consistently showed the strongest response to combination therapy across all markers tied to NVU integrity (Figure S6F). Overall, these findings indicate that combination therapy played a significant role in protecting the retina. It was also observed that INSs.c alone had no significant effect on these markers.

### Combination therapy modulates transcription factors and mitigates chronic retinal inflammation

**Figure 4:**
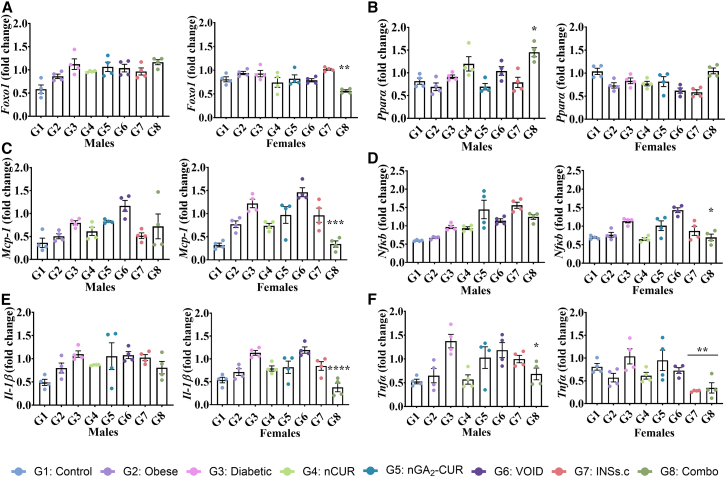
Combination therapy modulates transcription factors and mitigates chronic retinal inflammation (A and B) Real-time PCR analysis of mRNA levels for key transcription factors: (A) *Foxo1* and (B) *Pparα*. (C–F) Expression of pro-inflammatory markers: (C) *Mcp-1*, (D) *Nfκb*, (E) *Il-1β*, and (F) *Tnfα*. Data are normalized to the housekeeping gene *β-actin* and presented as fold change relative to controls (*n* = 4 biological replicates/group; 4 technical replicates for each sample). Data are presented as mean ± SEM. Data were analyzed using a one-way ANOVA followed by Tukey’s multiple comparison test for all-versus-all comparisons. False discovery rate (FDR) correction was applied to the dataset; all significant findings passed the FDR threshold (q < 0.05). Comparison annotations shown in the figure highlight differences between the diabetic group (G3) and combination group (G8). Full statistical comparisons are provided in Table S6. The significance levels used in the analysis were as follows: ∗*p* < 0.05, ∗∗*p* < 0.01, ∗∗∗*p* < 0.001, and ∗∗∗∗*p* < 0.0001. See also Figure S7 and Table S6.

Under INS resistance, oxidative stress and apoptosis are promoted by Forkhead box protein O1 (FOXO1).^41^ In diabetic mice, *Foxo1* expression was upregulated with no effect of treatment in males, whereas females showed a significant reduction with combination therapy (Figure 4A). Peroxisome proliferator-activated receptor alpha (*Pparα*), involved in anti-inflammatory and lipid metabolic functions,^42^ showed significant upregulation in the male combination group, suggesting protective effects whereas, females showed an upregulation but it was non-significant (Figure 4B). Effect size analysis showed moderate responses for both markers (*Foxo1*: ƞ^2^ = 0.61 males, 0.62 females; *Pparα*: ƞ^2^ = 0.69 males, 0.65 females), underscoring the relevance of these changes even when statistical significance was limited (Figures S7A and S7B).

Expression of inflammatory markers such as *Mcp-1, Nfκb, Il-1β,* and *Tnfα* was measured in retina (Figures 4C–4F). *Mcp-1* and *Nfκb* levels were significantly upregulated in diabetic females, whereas in males, *Mcp-1* was significantly upregulated only in the G6 group and in G5, G7, and G8 groups for *Nfκb*. In females, the combination therapy group showed a significant downregulation of these markers. The effect size was more pronounced in females (0.81) than males (0.56) for *Mcp*-1, while *Nfκb* responses were comparable across sexes (0.77 each) (Figures S7C and S7D). *Il-1β* expression increased in both sexes, yet females showed a more favorable response to the combination therapy, with a stronger treatment association (0.76) compared to males (0.43) (Figure S7 E).

*Tnfα*, a pro-inflammatory cytokine, was significantly upregulated in diabetic animals of both sexes and was effectively reduced by combination therapy in both, with a moderate effect size (ƞ^2^ = 0.60) (Figure S7F). Females receiving INSs.c alone also showed a notable decrease (Figure 4F).

When rank-ordering the data, females in G8 performed best, whereas in males, the G4 group fared better, followed by the G8 and G7 males (Figure S7G). This highlights a clear difference in treatment efficacy between sexes within this inflammatory panel, with females consistently showing the strongest response to the combination therapy.

### Preservation of vascular plexus integrity and attenuation of neuroinflammation

**Figure 5:**
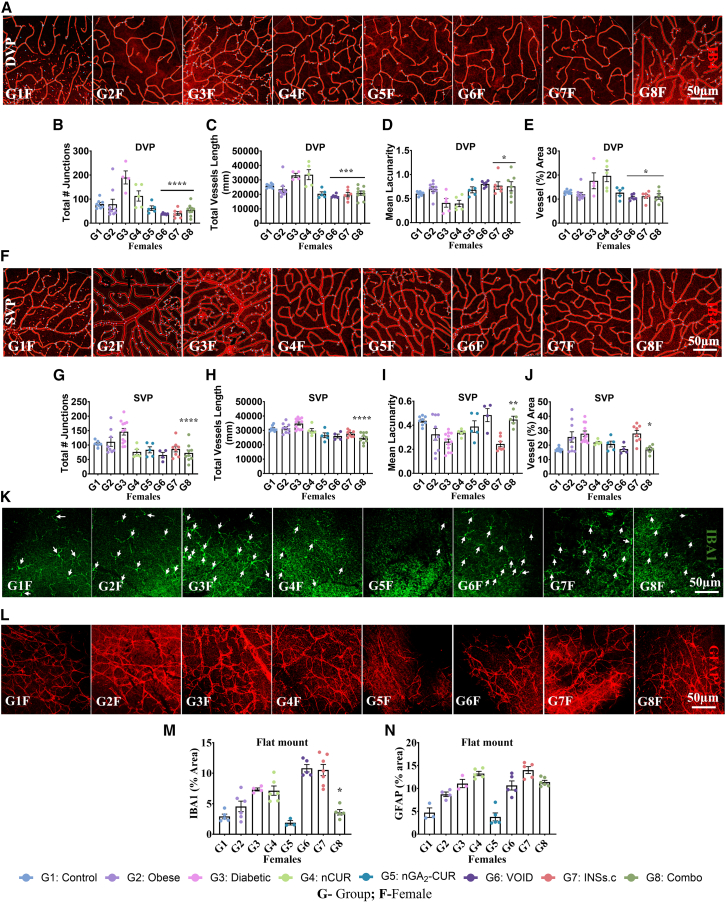
Preservation of vascular plexus integrity and attenuation of neuroinflammation (A and F) Representative AngioTool analysis of female retinal whole mounts stained with IB4 for (A) deep vascular plexus (DVP) and (F) superficial vascular plexus (SVP). The vascular skeleton is reconstructed in red, with branching points indicated in blue. (B–E) Quantification of DVP parameters: (B) Total number of junctions, (C) Total vessel length (mm), (D) Mean Lacunarity, and (E) Vascular area percentage. (G–J) Quantification of SVP parameters: (G) total number of junctions, (H) total vessel length (mm), (I) mean lacunarity, and (J) vascular area percentage. (K and L) Representative confocal images of female retinal whole mounts immunostained for (K) microglia (IBA1, white arrows) and (L) astrocytes (GFAP). (M and N) Quantification of positive area percentage for (M) IBA1 and (N) GFAP. Images were acquired using a confocal microscope (20x magnification; scale bar = 50 μm). Vascular parameters were quantified using AngioTool; glial area was quantified using ImageJ. Data are presented as mean ± SEM. Analysis was performed on *n* = 2 biological replicates per group (with multiple technical fields of view analyzed per retina, totaling 4–13 images per group). Data were analyzed using a one-way ANOVA followed by Tukey’s multiple comparison test for all-versus-all comparisons. False Discovery Rate (FDR) correction was applied to the dataset; all significant findings passed the FDR threshold (*q* < 0.05). Comparison annotations shown in the figure highlight differences between the Diabetic group (G3) and Combination group (G8). Full statistical comparisons are provided in Table S7. The significance levels used in the analysis were as follows: ∗*p* < 0.05, ∗∗*p* < 0.01, ∗∗∗*p* < 0.001, and ∗∗∗∗*p* < 0.0001. See also Figure S8 and Table S7.

Fundoscopy revealed vascular abnormalities such as pathological vascular remodeling and tortuosity in diabetics (G3), which were also observed in the G6 and G7 groups (Figure S8A), but less prominent in groups receiving CUR. Quantitative analysis of the vascular plexus revealed structural alterations indicative of early diabetes-related damage, distinct from classic DR pathology. These changes were particularly pronounced in the deep vascular plexus (DVP), a capillary bed known to exhibit earlier sensitivity to diabetic hypoxic stress compared to the superficial layers.^43^^,^^44^ To characterize these specific microvascular alterations, the DVP and superficial vascular plexus (SVP) were visualized in female retinal flat mounts using Isolectin B4 (IB4) staining (Figures 5A–5F; S8B). Diabetic mice (G3) showed increased vessel density and length, and reduced lacunarity, representing early changes which are further corroborated by the fundoscopy images and gene expression of angiogenic markers in retina (*Vegfa*, *Vegfr2*) presented earlier (Figures 5A–5J). Treatment with nGA_2_-CUR (G5, G8) and INSs.c (G7) reduced these pathological remodeling indices, with the most pronounced and consistent structural improvement with combinational therapy for all indices in both DVP and SVP.

To evaluate neuroinflammation, retinal flat mounts were stained with Ionized calcium-binding adapter molecule 1 (IBA1) (microglial/ macrophage marker) (green) and GFAP (macroglial cell marker) (red) (Figures 5K and 5L). The positive area for these markers are significantly higher in G3, which was significantly reduced in the G5 group. IBA1 showed a significant reduction in the G8 group but no significant change for GFAP expression (Figure 5M-N). Together, these findings demonstrate that the nGA_2_-CUR mitigate both the vascular and glial changes of early DR.

### Prevention of cataract progression and preservation of lens protein structural integrity

**Figure 6:**
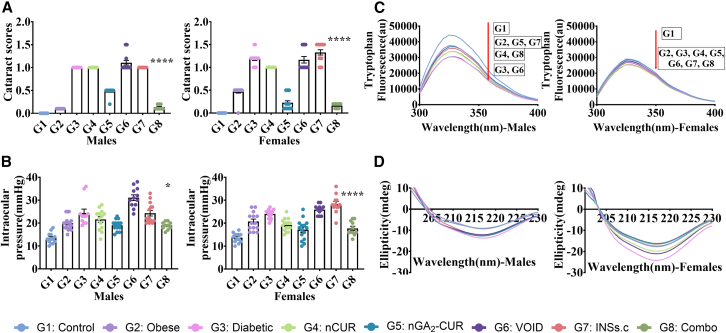
Prevention of cataract progression and preservation of lens protein structural integrity (A) Cataract severity scores assessed in the terminal week of the study (*n* = 14–16 eyes/ group). (B) Intraocular pressure (IOP) measurements recorded using a tonometer in the terminal week (*n* = 14–16 eyes/ group). (C) Far-UV circular dichroism (CD) spectra of lens homogenates, indicating changes in protein secondary structure (*n* = 7–8 eyes/ group). (D) Intrinsic tryptophan fluorescence intensity, reflecting alterations in protein tertiary structure and conformational stability (*n* = 7–8 eyes/ group). Data are presented as mean ± SEM. Data were analyzed using a one-way ANOVA followed by Tukey’s multiple comparison test for all-versus-all comparisons. Comparison annotations shown in the figure highlight differences between the diabetic group (G3) and combination group (G8). Full statistical comparisons are provided in Table S8. The significance levels used in the analysis were as follows: ∗*p* < 0.05, ∗∗*p* < 0.01, ∗∗∗*p* < 0.001, and ∗∗∗∗*p* < 0.0001. See also Figure S9 and Table S8.

Terminal cataract scores showed that diabetic mice in the G3 group had significantly higher scores, indicating the severity of lens cloudiness. The combination therapy (G8) and nGA_2_-CUR (G5) halted cataract progression in both sexes, yielding scores comparable to those of healthy lenses (Figure 6A). These scores were corroborated by representative slit lamp images (Figure S9A), which showed opacification (cloudiness) dots in G3 and G6 mice, while treatment with nGA_2_-CUR (G5 and G8) resulted in clear lenses similar to healthy controls. IOPs showed a trend similar to cataract scores, with significant reductions observed in the G5 and G8 groups (Figure 6B).

Spectroscopic analyses were conducted using circular dichroism (CD) and tryptophan fluorescence to evaluate lens protein integrity (Figures 6C and 6D). Lens crystallin proteins rely on their stable β-sheet secondary structure to maintain transparency. Any denaturation of this protein can lead to opacification of lens.^45^ Far-UV CD was utilized to determine changes in the secondary structure (peptide backbone) of the proteins.^46^ CD spectra from control animals showed a strong negative band at 217 nm, indicating stable β-strand structure. In diabetic males (G3), spectra were remarkably similar to those of controls (G1), preserving this negative band. In contrast, diabetic females exhibited a slightly broader band between 210 nm and 220nm with increased negative ellipticity, suggestive of secondary structural disruption and random coil formation.^46^^,^^47^

Intrinsic tryptophan fluorescence emission of lens homogenate was assessed to detect changes in protein tertiary structure (side-chain environment). When the lens is exposed to hyperglycemia, the tryptophan undergoes oxidation, leading to a change in intensity of the emission spectra in diabetic males (G3). Despite their intact CD spectra, the fluorescence intensity was reduced compared to controls, consistent with structural disruption. This dissociation of perturbed tertiary structure (fluorescence) alongside preserved secondary structure (CD), suggests that crystallin proteins in diabetic males undergo initial tertiary loosening and oxidative quenching prior to the complete unfolding of the peptide backbone.^48^^,^^49^ In contrast, the fluorescence peaks in females showed less variation between groups, likely because the structural damage had already progressed to the secondary level (as seen in CD). These distinct spectroscopic signatures suggest a sex-dimorphic trajectory in cataractogenesis, wherein males appear to exhibit early-stage tertiary destabilization, whereas females progress more rapidly toward secondary structural collapse. Rank ordering confirmed that males were most responsive to combination therapy, while females responded equally to G5 and G8 (Figure S9B).

### Effects of treatment on cardiac lymphangiogenic and metabolic dysfunction markers

**Figure 7:**
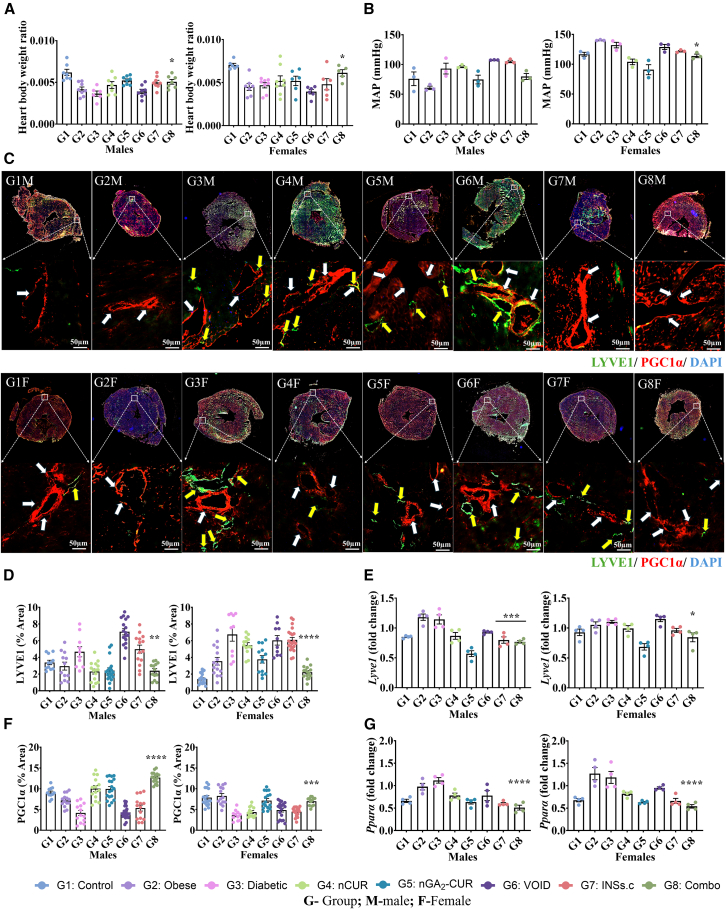
Effects of treatment on cardiac lymphangiogenic and metabolic dysfunction markers (A) Heart-to-body weight ratios. (B) Mean arterial pressure (MAP) measured via the tail-cuff method during the terminal study week (Week 25). (C) Representative immunofluorescence images of heart cryosections stained for LYVE1 (green), PGC1α (red), and DAPI (blue). Zoomed insets (scale bars, 50 μm) display marker distribution around major vessels, white arrows indicate blood vessels, and yellow arrows indicate lymphatic vessels surrounding the vasculature. (D and F) Quantification of (D) LYVE1 and (F) PGC1α positive staining area (*n* = 2–3 images from 5 to 6 mice/group). (E and G) Real-time PCR analysis of mRNA levels for (E) *Lyve1* and (G) *Pparα* in heart tissue. Data are normalized to *β-actin* and presented as fold change relative to controls (*n* = 4 biological replicates/group; 4 technical replicates for each sample). Images were acquired using a Zeiss Axioscan 7 microscope (20x magnification) and quantified via ImageJ. Data are presented as mean ± SEM. Unless otherwise noted, data were analyzed using a one-way ANOVA followed by Tukey’s multiple comparison test for all-versus-all comparisons. False Discovery Rate (FDR) correction was applied to the dataset; all significant findings passed the FDR threshold (*q* < 0.05). In females, the parameters Heart body weight ratio and MAP exhibited variance heterogeneity and were confirmed as significant discoveries by FDR analysis (*q* < 0.05). A pairwise analysis using Welch’s t-test identified significant differences between the diabetic and control groups (∗*p* < 0.05). All comparisons were made relative to the diabetic group (G3) and only the comparisons between Diabetic group (G3) and combination group (G8) are shown in figure. Additional comparisons are summarized in Table S9. The significance levels used in the analysis were as follows: ∗*p* < 0.05, ∗∗*p* < 0.01, ∗∗∗*p* < 0.001, and ∗∗∗∗*p* < 0.0001. See also Figure S10 and Table S9.

Early pathological diabetic cardiac remodeling is associated with dysfunctional lymphangiogenesis with impaired drainage and increased inflammation.^50^^,^^51^ Heart to body weight ratios were significantly increased in the G8 group (Figure 7A), with treatment impact being of moderate magnitude in males (ƞ^2^ = 0.50) and somewhat lower in females (0.41) (Figure S10A). To further characterize the structural impact of disease on cardiac geometry, we measured total transverse heart diameter (Figure S10G). In both sexes, diseased cohorts (G2, G3) exhibited a significant increase in heart diameter compared to control (G1), consistent with pathological cardiomegaly and ventricular dilation. The combination therapy (G8) effectively rescued this phenotype, resulting in a significant reduction in heart diameter in females and a prominent downward trend in males toward G1 baseline levels. MAP remained unchanged across male groups, whereas diabetic females (G3) exhibited a significant rise in MAP, which was effectively reduced in groups receiving CUR NPs (G4, G5, and G8) (Figure 7B). The effect size analysis showed that the therapeutic influence on stabilizing MAP was considerable (males ƞ^2^ = 0.75; females ƞ^2^ = 0.85), with females showing a more pronounced benefit, consistent with their higher susceptibility to diabetic complications (Figure S10B).

To evaluate the role of lymphatics in inflammation and cardiac metabolism, the lymphangiogenesis pathway (Lymphatic Vessel Endothelial Hyaluronan Receptor 1 (LYVE1)) and the metabolic PPARα-PGC1α pathway were further explored. The heart cryosections were immunostained with LYVE1 and peroxisome proliferator-activated receptor gamma coactivator 1-alpha (PGC1α) are represented in Figure 7C. Immunofluorescence for LYVE1 was reduced in the groups receiving the CUR-NPs, with the effect being significant across both sexes in the combination group (G8) (Figure 7D). The relative impact of treatment on LYVE1 immunostaining was moderate, with effect estimates of 0.59 in males and 0.64 in females (Figure S10C). These results were supported by *Lyve1* gene expression, which was increased in diabetic hearts (G3), indicating an early, probably compensatory response to diabetic cardiac changes. The nGA_2_-CUR groups (G5 and G8) were able to significantly decrease the levels of *Lyve1* expression across both sexes (Figure 7E). The large effect size of *Lyve1* gene expression (η^2^ = 0.84 in males, 0.72 in females) indicates that the therapy substantially reduced *Lyve1* expression (Figure S10D).

The metabolic regulation in the diabetic heart was assessed by quantifying the PGC1α-positive cells (Figure 7F) in heart cryosections and *Pparα* gene expression in heart tissues (Figure 7G). The area (%) for PGC-1α was significantly downregulated in diabetics indicating mitochondrial dysgenesis. It was significantly enhanced with ligand-coated NPs (G5 and G8), indicating the protective nature of these nGA_2_-CUR in both sexes (Figure 7F), with a moderate effect in males (η^2^ = 0.70) and low-to-moderate effect in females (η^2^ = 0.51) (Figure S10E). *Pparα* activation leads to impaired glucose utilization and abnormal lipid accumulation in cardiac tissue.^52^ It was seen that in both males and females, there was a significant upregulation of expression, indicating an increase in fatty acid oxidation in heart tissue in diseased animals. All treatment interventions were effective in downregulating the expression, but a significant difference was observed in the groups receiving combination therapy (G8) (Figure 7G). Effect estimates indicate robust treatment effects on *Pparα* gene expression in both males (0.76) and females (0.79), highlighting the therapy’s strong modulation of cardiac metabolic pathways (Figure S10F).

### Transcriptional regulation of key lymphangiogenic signaling markers

**Figure 8:**
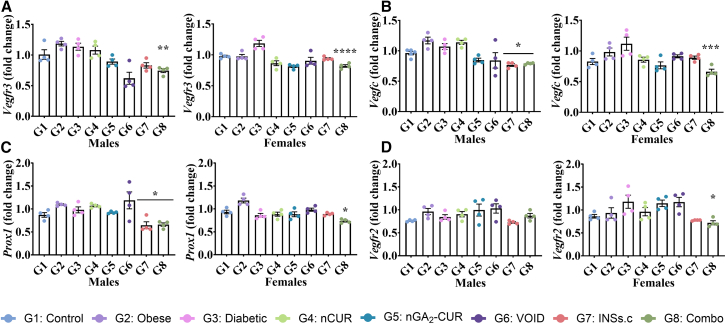
Transcriptional regulation of key lymphangiogenic signaling markers (A–D) Real-time PCR analysis of mRNA levels for early lymphangiogenesis markers: (A) *Vegfr3*, (B) *Vegfc*, (C) *Prox1*, and (D) *Vegfr2*. Data are normalized to the housekeeping gene *β-actin* and presented as fold change relative to controls (*n* = 4 biological replicates/group; 4 technical replicates for each sample). Data is presented as mean ± SEM. Unless otherwise noted, data were analyzed using a one-way ANOVA followed by Tukey’s multiple comparison test for all-versus-all comparisons. False discovery rate (FDR) correction was applied to the dataset; all significant findings passed the FDR threshold (*q* < 0.05). In both male and female cohorts, *Prox1* data exhibited variance heterogeneity. Consequently, statistical significance was determined using pairwise Welch’s t-tests (accounting for unequal variances), which identified significant differences between the diabetic and control groups (∗*p* < 0.05). All comparisons were made relative to the diabetic group (G3), and only the comparisons between diabetic group (G3) and combination group (G8) are shown in the figure. Additional comparisons are summarized in Table S10. The significance levels used in the analysis were as follows: ∗*p* < 0.05, ∗∗*p* < 0.01, ∗∗∗*p* < 0.001, and ∗∗∗∗*p* < 0.0001. See also Figures S11 and S12 and Table S10.

To analyze the progression toward pathological cardiac remodeling, the expression of early lymphatic development markers such as *Vegfr3, Vegfc,* and *Prox1* was assessed in the heart tissue, which are essential for proliferation, migration, and sprouting of lymphatic endothelial cells (Figures 8A–8C). Even though *Vegfr2* is associated with angiogenesis, it plays a role in the early development of lymphatics (Figure 8D). Overall, the gene expression of the lymphatic endothelial markers (*Vegfr3*, *Prox1*), and genes involved in lymphangiogenic signaling (*Vegfc*) was observed to be upregulated in diabetic and obese animals, especially in females. Combination therapy was most effective at reducing their expression across both sexes, indicating initial compensatory responses were mitigated. Effect size analysis indicated that the treatment had robust effects on *Vegfr3* (male: 0.76; female: 0.78), *Prox1* (male: 0.65; female: 0.75), and *Vegfc* (male: 0.70; female: 0.63) (Figures S11A–S11C). *Vegfr2* gene expression was downregulated in females in the combination and INSs.c groups but unchanged in males (Figure 8D), with low-to-moderate effect sizes (male: 0.40; female: 0.56) (Figure S11D). The data indicate that compensatory upregulation of lymphangiogenic activity in diabetic (G3) animals was effectively curtailed in the combination group (G8), followed by the G5 group. When ranked for these specific lymphangiogenic markers, combination therapy was most successful in females. In males, the INSs.c group scored lowest, closely followed by the combination group (Figure S11E).

Interestingly, genes involved in lipid metabolism (*Fas, Cpt1*) and INS signaling (*Irs1*) did not show any difference between control and diseased animals in both sexes (Figures S12A–S13C). For evaluating inflammatory signaling, *Stat3, Txnip, Tnfα, Tgfβ,* and *Nlrp3* gene expression was also evaluated in heart tissue (Figure S12D–S12H). There was not any upregulation of any inflammatory markers in male diabetic animals throughout the panel. In females, a significant upregulation of these inflammatory markers was observed in diabetic animals, which was downregulated significantly by the combination therapy across the inflammatory panel, indicating female diabetic mice were experiencing early inflammatory changes, unlike male mice. Figure S12I, gives a schematic picture of common mechanisms involved in the progression of DR and DCM.

### Pathological correlation analysis of systemic blood pressure and intraocular pressure

**Figure 9:**
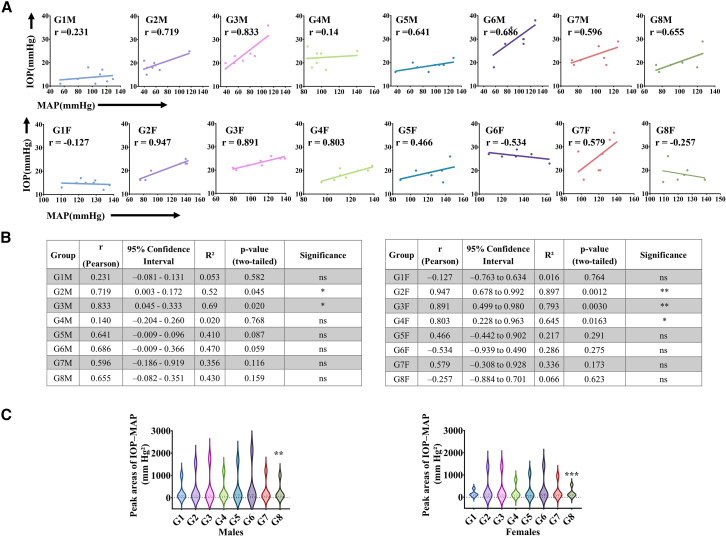
Pathological correlation analysis of systemic blood pressure and intraocular pressure (A) Correlation analysis of intraocular pressure (IOP) versus mean arterial pressure (MAP) across all experimental groups (G1–G8). Top row displays male cohorts; bottom row displays female cohorts. Dashed lines indicate the linear regression fit for each group. (B) Statistical summary of Pearson’s correlation analysis for key representative groups, detailing the correlation coefficient (r), 95% confidence interval (CI), coefficient of determination (R^2^), and *p* value. (C) Violin plots illustrate the distribution of area under the curve (AUC) derived from the IOP-MAP relationship. The AUC serves as a quantitative composite index of the overall pressure burden for each group. Pearson’s correlation coefficients (r) were calculated based on biological replicates (*n* = 7–8 mice per group). Significance levels indicate the strength of the linear relationship (B) or differences in AUC distribution relative to controls (C): ∗*p* < 0.05, ∗∗*p* < 0.01, ∗∗∗*p* < 0.001, and ∗∗∗∗*p* < 0.0001.

To determine if the observed ocular changes were driven by systemic hemodynamic instability, we analyzed the correlation between MAP and IOP across all groups (Figure 9A&B). In healthy control animals, no significant linear correlation was observed between MAP and IOP. This disconnect is consistent with intact ocular autoregulation, a physiological mechanism that buffers the eye against acute systemic pressure fluctuations to maintain stable perfusion.^53^

In contrast, diabetic mice exhibited a strong, statistically significant positive correlation between MAP and IOP, indicating a breakdown of these autoregulatory mechanisms. This finding aligns with established models of diabetic pathology, where early impairment of the NVU leads to a “pressure-passive” state, allowing systemic pressure to transmit directly to the intraocular compartment.^54^ This pathological vascular coupling was more pronounced in females (r = 0.891, *p* = 0.003) compared to males (r = 0.83, *p* = 0.02), suggesting a sex-specific vulnerability to the loss of vascular tone control. A similar pattern was observed in the G2 group.

Notably, treatment groups, particularly the G8, demonstrated a restoration of the physiological independence of IOP from MAP. In these groups, the linear relationship was abolished, mimicking the autoregulatory capacity seen in G1.

To quantify the overall magnitude of this pressure coupling, the AUC was calculated (Figure 9C). Reflecting the correlation data, AUC values were significantly elevated in diabetic and obese animals compared to controls, signifying a greater cumulative influence of systemic pressure on the eye. This burden was significantly higher in females. Treatment with the combination therapy effectively reduced the AUC to levels comparable to healthy controls, confirming the restoration of physiological vascular control.

## Discussion

Managing T2DM and its complications is challenging, as current therapies often target isolated pathways rather than addressing the systemic nature of the disease and are thus insufficient to prevent widespread, inflammation-driven organ damage. In this study, we tested a combination strategy using nGA_2_-CUR with INSs.c to enhance anti-inflammatory and antihyperglycemic effects, aiming to protect the eye and heart from diabetic complications. By improving CUR delivery and tissue targeting, the nGA_2_-CUR NPs demonstrated superior therapeutic benefit over non-functionalized NPs or INSs.c monotherapy alone. While previous studies in type 1 diabetic rats showed that nGA_2_-CUR with INSs.c could alleviate diabetic complications,^16^^,^^17^^,^^18^ the current study utilized the HFD/STZ-induced obese T2DM mouse model. This model allowed us to study both systemic metabolic dysfunction and tissue-specific complications, as it closely mimics human disease progression.

This study highlights significant sex differences in complication development. Males developed T2DM earlier with more severe hyperglycemia and inflammation, whereas females progressed more rapidly to complications such as cataract, DR, and cardiac dysfunction. Both sexes benefited from the combination therapy, though the therapeutic responses were heterogeneous. While females exhibited a more robust response across the inflammatory and neurovascular panels, males demonstrated strong, and sometimes superior, responsiveness in the prevention of structural protein denaturation (cataractogenesis) and cardiac metabolic regulation. This finding demonstrates that biological sex is an important determinant of disease outcome and emphasizes the critical need to include both sexes in mechanistic studies as a biological variable.

A major finding of this study is that it provides *in-vivo* physiological evidence for concomitant retinal-cardiac injury, where complications progress in parallel through shared molecular and cellular pathways. Our findings suggest that systemic and local inflammation, endothelial dysfunction, and mitochondrial/metabolic dysregulation act as common drivers across both tissues.

Systemic inflammation was evidenced by elevated acute-phase proteins (fibrinogen), adipokines (adipsin), and chemokines (RANTES and MIG). In the retina, glial activation (GFAP, IBA1) and pro-inflammatory mediators (*Mcp-1*, *Tnfα*, *Nfκb*) promoted neuroinflammation and disruption of the NVU. These same mediators are implicated in immune cell infiltration, fibroblast activation, and early fibrosis in the diabetic heart,^55^ showing a mechanistic overlap. Interestingly, while males showed early but less advanced cardiac inflammatory changes, females displayed a heightened inflammatory response (*Stat3, Tnfα, Tgfβ, Nlrp3*). These markers indicate that systemic inflammation underlies and drives the local tissue inflammation, leading to tissue injury in both the retina and heart. Combination therapy significantly suppressed both systemic and local inflammation, highlighting its broad anti-inflammatory effect.

Pathological vascular remodeling and endothelial dysfunction represented a second shared pathway. The retina showed increased levels of markers consistent with pathological angiogenesis and vascular leakage (*Vegfa, Vegfr2, Pdgfrβ*, IB4), while the heart showed increased lymphangiogenic signaling (*Lyve1, Vegfr3, Vegfc, Prox1, Vegfr2*). This indicates that while both tissues undergo pathological vascular remodeling, they do so through distinct but related VEGF/VEGFR-driven signaling pathways. Combination therapy significantly reduced these markers, preserved vascular integrity, and delayed early cardiac remodeling.

Metabolic and mitochondrial dysfunction was the third shared mechanism. In the retina, the upregulation of *Hif1α* and *Redd1* suggested hypoxic stress and altered stress signaling, while altered expression of *Pparα*, *Rbp3*, and *Aqp4* suggested a loss of neuro-protective functions. In the heart, key regulators of mitochondrial function, lipid metabolism, and energy balance (*Pparα*, PGC1α), were dysregulated. Additional metabolic markers measured in the heart (*Fas*, *Cpt*, *Irs1*) did not show significant differences at this stage of disease. Overall, these changes underscore that mitochondrial and metabolic dysfunction are central to both tissues, though with tissue-specific adaptations. Importantly, these findings suggest that retinal neurovascular changes are more advanced compared to early pathological cardiac remodeling, thus supporting the hypothesis that retinal degeneration may precede and predict early cardiac injury. These pathological changes were effectively attenuated with the combination therapy.

Beyond identifying the shared molecular pathways, we demonstrated the physiological consequences of this shared pathology. Our analysis of the relationship between MAP and IOP revealed that T2DM causes a pathological correlation, indicating breakdown of protective ocular autoregulation. This loss of vascular tone was more pronounced in females, consistent with their higher susceptibility to developing complications. The combination therapy was able to restore physiological function, consistent with its ability to protect anatomical structures and normalize the underlying molecular markers. This pathological relation likely arises from shared endothelial dysfunction, oxidative stress, and inflammatory signaling, thus establishing a physiological hemodynamic association under metabolic stress.

In conclusion, ocular complications may serve as an early physiological indicator for cardiac injury, reflecting shared mechanisms of inflammation, angiogenesis/lymphangiogenesis, and metabolic/ mitochondrial dysfunction. Treatment with dual-ligand CUR NPs in combination with INSs.c. effectively attenuated these pathways, thereby delaying cataract formation, preserving retinal neurovascular integrity, and protecting against early pathological cardiac remodeling. Notably, the functionalized NPs demonstrated these protective effects across systemic, cardiac, and ocular tissues at half the dosage of non-targeted controls (20 vs. 40 mg/kg). This validates the dose-sparing potential of the delivery system to minimize systemic exposure. This study highlights critical sex-specific differences and underscores the potential for multi-targeted nanotherapeutic strategies to address the concomitant progression of ocular and cardiovascular complications.

### Limitations of the study

We acknowledge certain limitations in our study design. First, the investigators were not blinded to the treatment groups during data collection and analysis, which presents a potential risk of bias. To mitigate this, we randomly allotted contralateral eyes to downstream assays (histology vs. molecular analysis) to prevent selection bias at the tissue level. Furthermore, we prioritized objective, quantitative readouts over subjective scoring by utilizing automated software algorithms for vascular analysis (AngioTool) and standardized thresholding for gene expression and quantitative immunofluorescence.

Second, while our model recapitulates key features of T2DM, the duration of the study represents an early-to-moderate stage of disease. Therefore, longer-term studies are necessary to evaluate the durability of the therapeutic effect on advanced complications. Future investigations will incorporate blind, randomized study designs and extended timelines to rigorously validate these translational findings. Additionally, while empty NPs (VOID) were used to control for the carrier material and formulation process, a separate vehicle-only control (solvent without polymer) was not included in the study design.

The electroretinography (ERG) to measure retinal electrical activity and echocardiography (Echo) or electrocardiography (ECG) to assess cardiac mechanical function would help to definitively map the mechanical and electrical severity of this systemic disease axis. While the absence of these specialized functional assays is a limitation, our study design prioritized the examination of the long-term, interconnected network of tissues over a 25-week period. The technical difficulty of maintaining a longitudinal functional cohort of this scale, comprising 8 experimental groups (∼*n* = 8 mice per group) is substantial. Specifically, the cumulative physiological stress of repeated anesthesia required for serial ERG and Echo measurements over six months can increase attrition rates and introduce confounding metabolic shifts in aging mice, potentially compromising the stability of the 64-animal cohort. In this context, our structural data, specifically Müller cell GFAP upregulation and transverse heart diameter, serve as validated surrogates for neural signaling failure and ventricular dilation. We believe these markers provide compelling evidence to characterize physiological relationships and tissue remodeling as they manifest specifically within this extended chronic setting. Future studies utilizing smaller, targeted cohorts in larger species may allow for the integration of continuous telemetry and serial functional assays, building upon structural frameworks established here to further map the real-time electrical kinetics of this disease axis.

Finally, for the analysis, rigorous family-wise error rate (FWER) corrections (Tukey) were applied within each individual experimental analysis to control for multiple pairwise comparisons between groups, while false discovery rate (FDR) adjustments were applied to the extensive biomarker panels. Despite these corrections, the broad scope of molecular markers assessed implies a potential risk of cumulative Type I error. Consequently, the molecular changes reported here should be interpreted as mechanistic, hypothesis-generating insights that provide a biological context for the robust phenotypic efficacy (e.g., cataract prevention, IOP normalization) observed. Furthermore, while this study focused on biological sex within a controlled murine model, animal models cannot account for the distinct gender-related, environmental, or lifestyle variables that influence human T2DM. Future translational studies will be required to validate these sex-specific nanotherapeutic outcomes in clinical cohorts.

## Resource availability

### Lead contact

Requests for further information and resources should be directed to and will be fulfilled by the lead contact, M.N.V.R. Kumar (mnvrkumar@ua.edu).

### Materials availability

This study did not generate new unique reagents.

### Data and code availability

• Raw data for Figures 1 and 8 have been deposited at Mendeley and are publicly available as of the date of publication at Mendeley Data: https://doi.org/10.17632/5tvzcbrf6y.1.
• This paper does not report original code.
• All data needed to evaluate the conclusions in the paper are present in the paper and/or the Supplementary Materials. Any request for further information and resources is available from the lead contact upon request.

## Acknowledgments

This work is supported by the 10.13039/100000002National Institutes of Health, United States (grant no. R01EY028169).

## Author contributions

S.R.A.: methodology, investigation, formal analysis, and writing – original draft; R.G.: writing (review and editing), supervision, project administration, methodology, investigation, and formal analysis; M.A.: polymer synthesis and formulation, writing (review and editing), supervision, project administration, methodology, investigation, and formal analysis; S.I.: investigation; S.D.: investigation; R.F.: writing (review and editing); R.B.: writing (review and editing); M.N.V.R.K.: writing (review and editing), supervision, resources, project administration, methodology, investigation, funding acquisition, and conceptualization.

## Declaration of interests

The authors declare no competing interests.

## STAR★Methods

### Key resources table

**Table:** 

REAGENT or RESOURCE	SOURCE	IDENTIFIER
**Antibodies**		

HIF1α	Invitrogen	Cat# MA1-16504; RRID: AB_568567
GFAP	Invitrogen	Cat#PA5-1629; RRID: AB_10980769
Goat anti-Mouse IgG Secondary Antibody Alexa Fluor™ 555	Invitrogen	Cat#A21425; RRID: AB_2535846
Goat anti-Rabbit IgG Secondary Antibody Alexa Fluor™ 488	Invitrogen	Cat#A11070; RRID: AB_2534114
ProLong™ Gold Antifade Mountant with DNA Stain DAPI	Invitrogen	Cat#P36935;RRID: SCR_015961
Lycopersicon esculentum (tomato) Lectin-LEA, DylightTM 594	Invitrogen	Cat#L32471
IBA1	Abcam	Cat#AB153696; RRID: AB_2889406
Alexa Fluor 594 F(ab’)2 fragment of goat anti-rabbit IgG	Invitrogen	Cat#A11072; RRID: AB_142057
AF488 conjugated LYVE1	Cell Signaling	Cat#69240S
PGC1α	Santa Cruz	Cat#sc-518025; RRID: AB_2890187

**Chemicals, peptides, and recombinant proteins**		

Curcumin	Acros Organics	Cat#AC169110050
Streptozotocin	Sigma-Aldrich	Cat#S0130
Gambogic Acid	Broadpharm	Cat#BP-10313
Lantus	Sanofi-Aventis	Cat#NDC 0088-2220-33
PLGA 50:50 (Resomer 503H)	Evonik	Cat#713296
1-Ethyl-3-(3-dimethylaminopropyl) carbodiimide (EDC)	Oakwood Chemicals	Cat#043232
Tris(2-aminoethyl) amine (TREN)	Oakwood Chemicals	Cat#007122
High Fat Diet	Research Diets	Cat#D12492
Low-Fat Control Diet	Research Diets	Cta#D12450K

**Critical commercial assays**		

Quick Start™ Bradford Protein Assay	Bio-rad	Cat#5000201
RNeasy Plus Universal Mini Kit	Qiagen	Cat#73404
iScript™ Advanced cDNA Synthesis Kit	Bio-rad	Cat#1725038
OCT medium	Leica Biosystems FSC Blue	Cat#NC0278476
Mouse Cytokine/ chemokine Magnetic Bead Panel	Millipore Sigma	Cat#8MCYTMAG-70K-PX32
Mouse Acute Phase Magnetic Panel 2 kit	Millipore Sigma	Cat#8MAP2MAG-76K
Mouse Fibrinogen ELISA Kit	Crystal Chem	Cat#80637

**Deposited data**		

Raw and analyzed data	This paper; Mendeley Data	Mendeley Data: https://doi.org/10.17632/5tvzcbrf6y.1

**Experimental models: Organisms/strains**		

C57BL/6J	The Jackson Laboratory	Stock#000664; RRID: IMSR_JAX:000664; Table S1

**Oligonucleotides**		

PCR primers	Integrated DNA Technologies	Table S2

**Software and algorithms**		

Zen blue 3.10 software	Carl Zeiss Microscopy GmbH	https://www.zeiss.com/microscopy
ImageJ	NIH	https://imagej.nih.gov/ij/
GraphPad Prism 10.1.2	GraphPad Software	https://www.graphpad.com/
Angiotool	NIH	https://ccrod.cancer.gov/wiki-html/ROB2/index.html

**Other**		

Alphatrak 3 Glucometer	Zoetis	
CODA® High Throughput System	Kent Scientific	
Pictor Plus™ Fundus Camera	Volk Optical	
iVivo® Funduscope	OcuScience	
Kowa SL-17 Portable Slit Lamp	Kowa American Corporation	
Tonometer	Icare tonolab	
Chirascan V100 circular dichroism spectrometer	Applied Photophysics	
BioTek Cytation 5	Agilent	
Bio-Plex 200 System	Bio-rad	
CFX Opus 384 RT-PCR system	Bio-rad	
UV Cryostat	Leica CM 1860	
AxioScan7 slide scanner	Zeiss	
LSM-900 Confocal Microscope	Zeiss Microscopy	

### Experimental model and study participant details

#### Experimental animals

*In vivo* studies were conducted using wild-type C57BL/6J mice. A total of 128 mice (64 males (M) and 64 females(F)) were obtained from the Jackson Laboratory (Bar Harbor, ME; Stock #000664). The study was initiated when the animals were 8 weeks of age. Biological sex was integrated as a primary experimental variable to assess sex-specific disease progression and therapeutic efficacy. The male and female cohorts were powered equally and analyzed independently.

#### Maintenance and care

Animals were closely monitored in a controlled environment maintained on a 12-hour light/dark cycle throughout the 25-week study period. Mice were housed in well-ventilated cages (maximum of 4 mice per cage) with environmental enrichment. Male and female mice were housed separately. Throughout the study, animals had *ad libitum* access to water and their designated diets (Low-Fat control diet or High-Fat diet, as specified in the experimental design) (Figure S2).

#### Institutional permission

All animal husbandry and experimental procedures were performed in strict accordance with institutional guidelines approved by the University of Alabama Institutional Animal Care and Use Committee (IACUC Protocol # 20-12-4214) and National Institutes of Health (NIH) guidelines.

Schematic experimental plan of the study is shown in Figure S2. Following a one-week acclimatization period, mice were assigned to specific dietary cohorts. Group 1 mice (G1: Control; n=16, 8M/F) were fed a Low-Fat diet (D12450K; Research Diets, New Brunswick, NJ). All other groups received a High-Fat diet (HFD, 60% kcal from fat; D12492; Research Diets). After 8 weeks of dietary manipulation, all animals except for those in Group 1 and Group 2 (G2: Obese; n=16, 8M/F), were given two intraperitoneal injections of Streptozotocin (STZ) (Sigma Chemicals, SLBJ7785V), dissolved in 0.1M citrate buffer (pH 4.5)^56^. The first dose of STZ (75mg/kg) was followed 3 days later by a second injection of 50mg/kg. Mice exhibiting non-fasting blood glucose levels >200 mg/dL were considered diabetic and eligible for study. All STZ-injected mice with hyperglycemia (blood glucose >200 mg/dL) were included in the study, and thus no animals were excluded from the study. To ensure balanced baseline characteristics, animals were stratified by blood glucose levels and assigned to groups so that the distribution of severe and moderate hyperglycemia was identical across all groups.

The animals received their respective treatments everyday morning for a period of 16 weeks as follows: Group 3 no treatment (G3: Diabetic; n=16, 8M/F). Group 4 received oral Curcumin (CUR)-loaded nanoparticles (NPs) (G4: nCUR; 40mg/kg/day CUR equivalent, n=16, 8M/F). For Group 5, received gambogic acid (GA)-ligand decorated NPs loaded with CUR (G5: nGA_2_-CUR; 20mg/kg/day CUR equivalent, n=16, 8M/F). The dosage was set to 20 mg/kg/day. This dose reduction for the targeted group was selected based on our previous pharmacokinetic studies demonstrating that this nanoparticle formulation enhances the oral bioavailability of curcumin by approximately 9-fold compared to native forms.^56^^,^^57^ Therefore, a lower dose was chosen for the targeted group to explicitly evaluate the dose-sparing efficacy of the GA-ligand modification. Group 6 received empty PLGA-GA_2_ NPs (G6: VOID; empty NPs CUR equivalent 20mg/kg, n=16, 8M/F) orally, Group 7 received subcutaneous insulin injections (G7: INSs.c.; insulin glargine 0.5IU/kg/day subcutaneously) and Group 8 received insulin injections along with oral gavage of nGA_2_-CUR suspension (G8: COMBO; insulin glargine 0.5IU/kg/day subcutaneously followed by oral gavage of 20mg/kg/day CUR equivalent GA decorated NPs, n=16, 8M/F) (Figure S2). Detailed information regarding animal allocation, attrition (mortality), and final group sizes is provided in Table S1.

Body weights were recorded biweekly. Blood glucose was measured biweekly by tail prick using a glucometer (Alphatrak 3, Zoetis). For the intervention groups (G4-G8), blood glucose was measured at two time points, one is prior to dosing (to establish a 24-hour profile) and one-hour post-dosing. To assess the cumulative glycemic burden over the study duration, the Area Under the Curve (AUC) was calculated for longitudinal blood glucose levels. This calculation was performed using the GraphPad Prism software. The analysis encompassed 13 time points measured biweekly from the start of study until the study endpoint. Blood pressure was recorded monthly by tail-cuff (CODA® High Throughput System).

After 16 weeks of treatment, animals were euthanized under non-fasted conditions during light cycle to minimize circadian variability. Blood was collected via cardiac puncture, and plasma was separated by centrifugation (3000 rpm, 30 min, 4°C). Major organs were harvested and stored at –80 °C or fixed in formalin.

### Method details

#### PLGA-GA_2_ synthesis and characterization

The reagents are listed in key resources table. All other chemicals unless otherwise stated, were obtained from Fisher Scientific (USA). The PLGA-GA_2_ was synthesized following established protocols with modifications for scale-up.^14^^,^^16^^,^^17^^,^^18^ Briefly, PLGA (50:50, Resomer 503H) was functionalized with tris(2-aminoethyl) amine (TREN) via carbodiimide-mediated coupling. Initially, TREN-diBoc was conjugated to PLGA using 1-ethyl-3-(3-dimethylaminopropyl) carbodiimide (EDC) chemistry. After purification via precipitation, the diBoc-protected TREN-PLGA intermediate was deprotected to yield free primary amine groups. These free amines were then used to conjugate GA in a second EDC-mediated coupling reaction, which resulted in the final PLGA-GA_2_ conjugate. Various spectroscopic techniques were used for confirmation of successful conjugation; NMR revealed the appearance of characteristic GA aromatic peaks in the range of 6.5–7.0 ppm confirmed the attachment of GA. Fourier-Transform Infrared (FTIR) Spectroscopy shown the evidence of amide bond, with a C=O stretching vibration peak appearing between 1670–1630 cm^-1^ and an N–H bending peak between 1650–1560 cm^-1^. The structural integrity of the polymer backbone and the success of synthesis were further validated by Gel Permeation Chromatography (GPC), which confirmed that the molecular weight distribution remained within acceptable limits after modification (Figures S1A–S1C).

#### CUR-loaded nanoparticles

CUR-loaded NPs were prepared on a large scale over approximately 60 batches using an oil-in-water (O/W) emulsification technique. Briefly, PLGA or PLGA-GA_2_ and CUR were dissolved in ethyl acetate and added dropwise to an aqueous PVA solution under stirring, followed by homogenization, solvent evaporation, centrifugation, and lyophilization. In brief, the organic phase was prepared by dissolving PLGA or PLGA-GA_2_ polymer (500 mg) and CUR (75 mg) in 25 mL of ethyl acetate. This solution was stirred at 1000 rpm for 1 hour to ensure complete dissolution. The resulting organic phase was then added dropwise to an aqueous phase containing 1% PVA (500 mg in 50 mL deionized water) under continuous stirring. The emulsion was stirred at 1500 rpm for 45 minutes, followed by homogenization at 15,000 rpm for another 45 minutes. To promote solvent evaporation, the emulsion was diluted with 200 mL of deionized water and stirred overnight at room temperature. The resulting emulsion was then centrifuged at 15,000 × g for 30 minutes at 4 °C. The pellet was resuspended in 25 mL of 5% (w/v) sucrose solution and stored at −80 °C prior to lyophilization. Freeze-drying was carried out using a benchtop freeze dryer (Labconco FreeZone Triad). Samples were lyophilized at −55 °C for 48 hours under a vacuum of 0.002 mbar, followed by a secondary drying step at 20 °C for 20 hours under the same vacuum conditions. The dried formulations were crimp-sealed and stored at 4 °C until further use in animal experiments. For the preparation of PLGA-GA_2_ (void) particles (i.e., without CUR), the same procedure was followed, excluding the addition of curcumin to the organic phase. The final dried formulations, including PLGA (nCUR), PLGA-GA_2_ (nGA_2_-CUR), and PLGA-GA_2_ (VOID), were later resuspended in appropriate media for characterization. Dynamic Light Scattering (DLS, Malvern) was used to determine particle size and polydispersity. Scanning Electron Microscopy (Apreo FE-SEM) was employed to assess morphology, and High-Performance Liquid Chromatography (HPLC) was used to calculate the entrapment efficiency of CUR (Figures S1D–S1F).

#### Ocular sampling and experimental units

For *in vivo* assessments (intraocular pressure measurements and cataract scoring), data were collected from both eyes of each animal (n=14-16 eyes/group). This approach allowed for the evaluation of potential intra-animal variability and asymmetry in disease progression. Following euthanasia, the eyes were enucleated for *ex vivo* analyses. One eye from each animal was utilized for molecular and biochemical assays, while the contralateral eye was allocated for histological assessment. Within the histology group, eyes were randomly divided into two subsets. One subset was processed for paraffin embedding and cross-sectioning (n=4), while the remaining eyes were prepared for retinal flat mount analysis (n=4). Therefore, for all *ex vivo* endpoints, the experimental unit represents the individual animal.

#### Slit Lamp Cataract Grading & Fundoscopy

The eye lens was examined biweekly using a Kowa SL-17 Portable Slit Lamp and lens images were captured using Pictor Plus™ Fundus Camera (Volk Optical, USA). Retinal fundoscopic examination was done using OcuScience iVivo® Funduscope. Ocular pressure was also measured using Icare tonolab. Before the examination, pupils were dilated using 1-2 drops of tropicamide ophthalmic solution, USP (1%). Initiation and progression of lens opacity were graded into five categories as described previously.^16^

#### Lens protein CD and tryptophan fluorescence

A 10% homogenate of lens were prepared in phosphate buffer (PBS), pH 7.4 using bead blaster (Benchmark). Homogenates were centrifuged at 13000 rpm for 15 min. The supernatant (soluble fraction) was collected and quantified using Quick Start™ Bradford Protein Assay (Bio-Rad; Cat #5000201) and a working solution of 0.5mg/ml in PBS was made for further analysis of lens proteins. Chirascan V100 circular dichroism spectrometer was used to measure Far-UV spectra of lens protein (n=6-8/group, scan range:180-300nm), under following conditions: response of 0.5s, 1.0 nm bandwidth, in a 0.5mm path length cell (100μL) at 25 °C. The spectra obtained were corrected by subtracting the spectra of the buffer, then averaged and smoothened. Water soluble (WS) 0.5 mg/ml lens protein samples (n=6-8/group) in PBS were analyzed by recording the fluorescence emission spectra (Cytation 5, BioTek, Agilent) for Tryptophan. In a 96-well plate, 100μL of aliquots were excited at 295±10 nm and emission monitored at 300–400 ±10 nm with expected peak length at 332nm and acquisitions of 10 scans per well. Procedures for lens protein extraction and spectroscopic analysis were carried out exactly as described in our previously established protocol, without modification.^16^

#### Multiplex and ELISA kits

Plasma analytes were analyzed using the Mouse Cytokine/ chemokine Magnetic Bead Panel (Millipore Sigma; Catalog ID: MCYTMAG-70K-PX32), and Mouse Acute Phase Magnetic Panel 2 kit (Millipore Sigma; Catalog ID: MAP2MAG-76K) on a Bio-Plex 200 System (Bio-Rad). Fibrinogen was measured using Mouse Fibrinogen ELISA Kit (Crystal Chem, Illinois; Cat# 80637). All assays were performed according to the manufacturer’s instructions.

#### RNA isolation and qPCR

RNA was isolated from retina and heart using RNeasy Plus Universal Mini Kit (Qiagen; Cat no. 73404) according to manufacturer instructions. RNA concentration and purity were checked using BioTek Cytation 5 (Agilent). The isolated RNA of four randomly selected animals per group (n=4 biological replicates) was used for cDNA synthesis with the iScript™ Advanced cDNA Synthesis Kit (BioRad; Cat #1725038) and was used for Real-time PCR amplification of target transcripts. Each biological sample was run in quadruplicate (four technical replicates) on a CFX Opus 384 RT-PCR system (BioRad, Hercules, CA) with PowerUp SYBR Green (Applied Biosystems; Cat no, A25742) as the fluorescent dye. The primers used in this study (Table S2) were pre-designed and validated by the Harvard Primer Bank and sourced from Integrated DNA Technologies (IDT). The resulting threshold cycle (C_T_) values were averaged for each animal. ΔC_T_ values were calculated by normalizing the average C_T_ of the target gene to the housekeeping gene, *β- actin*. Subsequently, ΔΔC_T_ values were derived by calibrating each sample to the mean ΔC_T_ of the diseased group. Relative fold change was calculated using the 2^∧^−ΔΔC_T_ method.

#### Retinal cross-sections

Formalin fixed paraffin embedded eyes were sectioned about 5 μm thickness. H&E staining was performed on paraffin sections as a standard procedure. These sections underwent immunofluorescence analysis to assess HIF1α and GFAP expression. The deparaffinized sections were steamed in 1X citrate buffer to retrieve the antigen for 15 minutes, followed by 2 hours blocking with 3% blocking buffer to minimize non-specific binding. Slides were incubated with primary antibodies targeting HIF1α (Invitrogen; Cat# MA1-16504) (1:50) and GFAP (Invitrogen; Cat# PA5-16291) (1:200) overnight at 4 °C. After 1X PBS washing, sections were incubated with secondary antibodies conjugated to Goat anti-Mouse IgG Secondary Antibody, Alexa Fluor™ 555 (Invitrogen; Cat# A21425) (1:1000) for HIF1α and Goat anti-Rabbit IgG Secondary Antibody Alexa Fluor™ 488 (Invitrogen; Cat# A11070) (1:1000) for GFAP in 1.5% blocking buffer; DAPI was used for nuclear staining. Slides were then mounted with coverslips with ProLong™ Gold Antifade Mountant with DNA Stain DAPI (Invitrogen; P36935). Images were acquired using a Zeiss AxioScan7 slide scanner, then processed using Zen blue 3.10 software and quantification were carried out using ImageJ.

#### Retinal flat mounts

Retina was isolated from formalin fixed mouse eyes as described by our lab previously.^58^ Then the retina was washed with PBS and permeabilized with 1% Triton-X-100 then incubated in 3% horse serum for 2 hours on a rocker. Followed by incubation with Lycopersicon esculentum (tomato) Lectin-LEA, DylightTM 594 (Invitrogen, Cat# L32471) (referred as IB4 hereafter), and primary antibodies IBA1 (Abcam, Cat # AB153696) and GFAP (Invitrogen, Cat# PA5-16291) rocking at 4°C. Secondary antibodies to Alexa Fluor 488 F(ab’)2 fragment of goat anti-rabbit IgG (Invitrogen, Cat# A11070) and Alexa Fluor 594 F(ab’)2 fragment of goat anti-rabbit IgG (Invitrogen, Cat# A11072) were added respectively to the primary antibodies and rocked for 2 hrs in dark. Then the retinas were flattened onto slide with inner retina facing coverslips and mounted with an antifade mounting medium (Invitrogen). Images were obtained using a confocal microscope (LSM-900, Zeiss Microscopy Germany).

#### Heart cryosections & immunofluorescence

The base of the heart, immediately after animal sacrifice, were embedded in OCT medium (Leica Biosystems FSC Blue, Cat # NC0278476) and were stored at -80°C until further processing. A subset of six embedded tissues per group was randomly selected for cryo-sectioning and immunofluorescence analysis. Using UV Cryostat (Leica CM 1860), a 5 μm thick section was made at -15°C onto adhesive slides (Leica white X-tra Slides, Cat # 3800200AE). The cryo-sections were treated with ice-cold acetone for fixation, then were washed with PBS 3 times and were incubated with 3% horse serum for 2 hours. Then the heart sections were incubated overnight at 4°C with AF488 conjugated LYVE1 (Cell Signaling, Cat # 69240S) and unconjugated PGC1α (Santa Cruz, Cat #sc-518025) antibodies at 1:50 dilution in 1.5% horse serum in PBS. Following primary antibody incubation, the slides were washed with PBS 3 times and were further incubated with goat anti-mouse IgG Alexa Fluor-555 secondary antibody (Invitrogen, Cat# A21425) against PGC1α at 1:1000 dilution for 2 hours. Following PBS washes, coverslips were mounted with ProLong™ Gold Antifade Mountant with DNA Stain DAPI (Invitrogen; P36935). Slides were scanned using a Zeiss AxioScan7 slide scanner, then quantified using Zen blue 3.10 and ImageJ software.

### Quantification and statistical analysis

The sample size was determined based on an *a priori* power analysis (G∗Power 3.1) to allow for independent statistical evaluation of both sexes (Sex as a Biological Variable). Based on historical data for STZ-induced diabetes models, we anticipated a large effect size (Cohen’s *d* ≥ 1.2) for the primary endpoint (blood glucose reduction). To achieve a statistical power (1-β) of 80% with a type I error rate (α) of 0.05, a minimum of n = 6 animals was required for each comparison. Therefore, the study was designed with n = 8 males and n = 8 females per group to ensuring that both sexes retained sufficient independent statistical power after accounting for potential attrition.

Statistical analyses were performed using GraphPad Prism software (version 10.1.2; GraphPad Software, San Diego, CA). All quantitative data are expressed as mean ± Standard Error of the Mean (SEM). To determine statistical significance among multiple groups, a one-way Analysis of Variance (ANOVA) was utilized. Post-hoc comparisons were conducted using Dunnett’s multiple comparison test when comparing treatment groups against the Diabetic control (G3), or Tukey’s multiple comparison test for all-versus-all group comparisons, as specified in the figure legends. For datasets exhibiting variance heterogeneity where the assumptions of homoscedasticity were violated, planned pairwise comparisons were assessed using Welch’s t-test (not assuming equal variances).

To ensure statistical rigor and control the family-wise error rate across the extensive biomarker panels, the Benjamini-Hochberg procedure was applied to control the False Discovery Rate (FDR) with a threshold of *Q* = 0.05 (5%). A *p*-value of < 0.05 was considered statistically significant. Comprehensive statistical evaluations for all analytes, including FDR-adjusted *q*-values, are provided in Table S11. Furthermore, to provide granular insight into these results, we have generated detailed tables of pairwise comparisons, which have been placed in the Supplementary Material (Tables S3 and S10).

Given the multi-group design and the complexity of the underlying pathological pathways, a composite rank-ordering system was employed to capture the overall relative efficacy of the treatments across the diverse spectrum of molecular and biochemical markers. This methodology provides a holistic assessment of therapeutic benefits, contrasting with isolated marker-by-marker analyses. Treatments were ranked from 1 to 5 for each individual marker, with a rank of 1 indicating the most effective outcome (closest to the healthy/therapeutic ideal) and 5 indicating the least effective among the intervention groups (G4–G8). The sum of the ranks was calculated for each group across all marker categories, with a lower total indicating greater cumulative efficacy.

The formula used to rank the data is given below:

$$\frac{Untreated\;Biomaker-Treated\;Biomarker}{Untreated\;Biomarker}\times 100$$

Furthermore, Quantile-Quantile (Q-Q) plots were utilized as a diagnostic tool for the raw data from all marker categories. Data normality was assessed using Q-Q plots, which helped to visually assess the distributional assumptions of the data, which informed the decision to use a rank-based, non-parametric approach to synthesize the final treatment efficacy scores. By converting the disparate quantitative changes of numerous markers into a single ordinal scale (the rank), the system achieves the following: a) it rewards treatments that demonstrate a consistent therapeutic effect across a broad range of interconnected pathways; b) it provides a clear, single metric of overall efficacy by synthesizing large, disparate datasets into a manageable, comparative total score; and c) it focuses on the relative performance of the five active treatments (G4–G8) among themselves and against the untreated diabetic control (G3). Additionally, biological effect size was evaluated by calculating Eta-squared (*ƞ*^*2*^), quantifying the proportion of total variance attributable to the treatment effect, thereby providing a measure of the magnitude of the therapeutic intervention independent of sample size.

Linear regression and the Pearson correlation coefficients analyses were performed to evaluate the relationship between IOP-MAP in groups (G1–G8). To assess the extent of correlation between ocular and systemic pressures, the peak area (AUC) was calculated from intraocular pressure (IOP) and mean arterial pressure (MAP) measurements obtained for each experimental group. Paired IOP and MAP values were collected from individual animals (n=7- 8 per group) and plotted as IOP (y-axis) versus MAP (x-axis) to generate pressure–pressure response curves. Each curve typically exhibited a single dominant peak corresponding to the maximal ocular response to systemic pressure variation.

## Supplemental information

Document S1. Figures S1–S12 and Tables S1–S8, S10, and S11

Table S9. Statistical Evaluation related to Figure 7
