# Supplementary material for: Sex-specific efficacy of curcumin-loaded nanoparticles and insulin for ocular-cardiac complications in type 2 diabetes mice
Source: iScience. 2026 Jul 2;29(7):116594. doi: 10.1016/j.isci.2026.116594 (PMC13355217; doi:10.1016/j.isci.2026.116594)
Supplement: Document S1. Figures S1–S12 and Tables S1–S8, S10, and S11 [file mmc1.pdf]

iScience, Volume *29*

Supplemental information

Sex-specific efficacy of curcumin-loaded nanoparticles and insulin for ocular-cardiac complications in type 2 diabetes mice

Swetha R. Allamreddy, Raghu Ganugula, Meenakshi Arora, Sabrina Ingram, Subhash Dwivedi, Richard Friend, Rita Basu, and M.N.V. Ravi Kumar

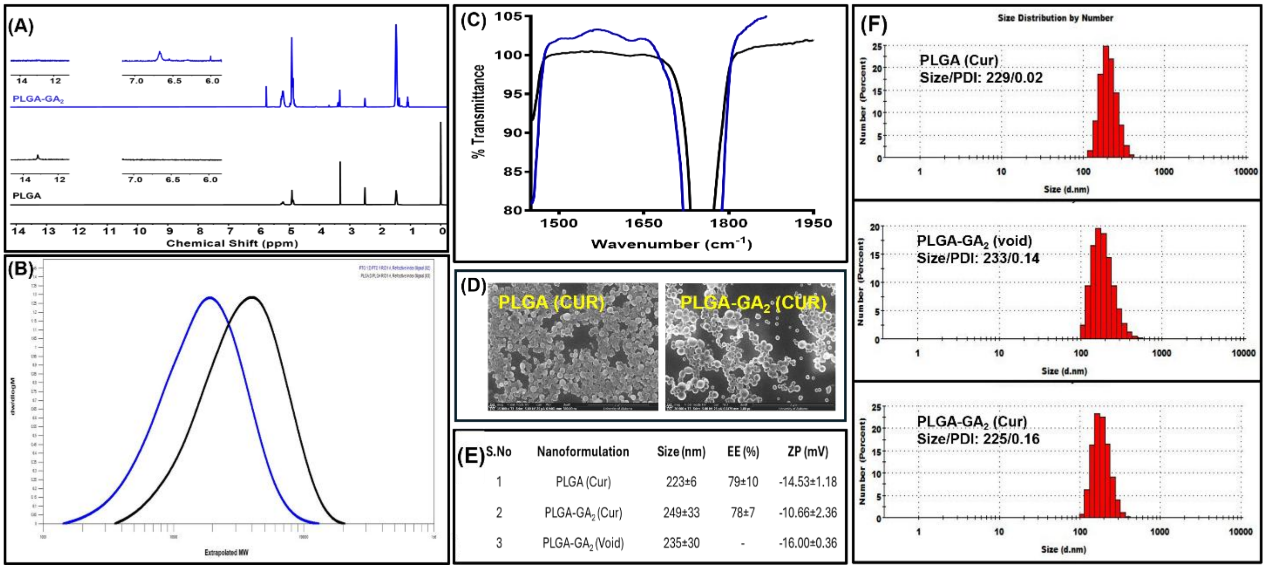

Figure S1: Details of polymer synthesis and nanoformulation preparations. Related to STAR Methods. A) ^{1}H NMR spectra of PLGA and PLGA-GA_2, B) GPC chromatograms of PLGA and PLGA-GA_2, C) FTIR spectra of PLGA and PLGA-GA_2, D) SEM pictographs of nanoformulations of PLGA and PLGA-GA_2, E) Pharmaceutical details of the nanoformulations and F) DLS spectrograph of nanoformulations PLGA (Cur), PLGA-GA_2 (Cur), and PLGA-GA_2 (Void).

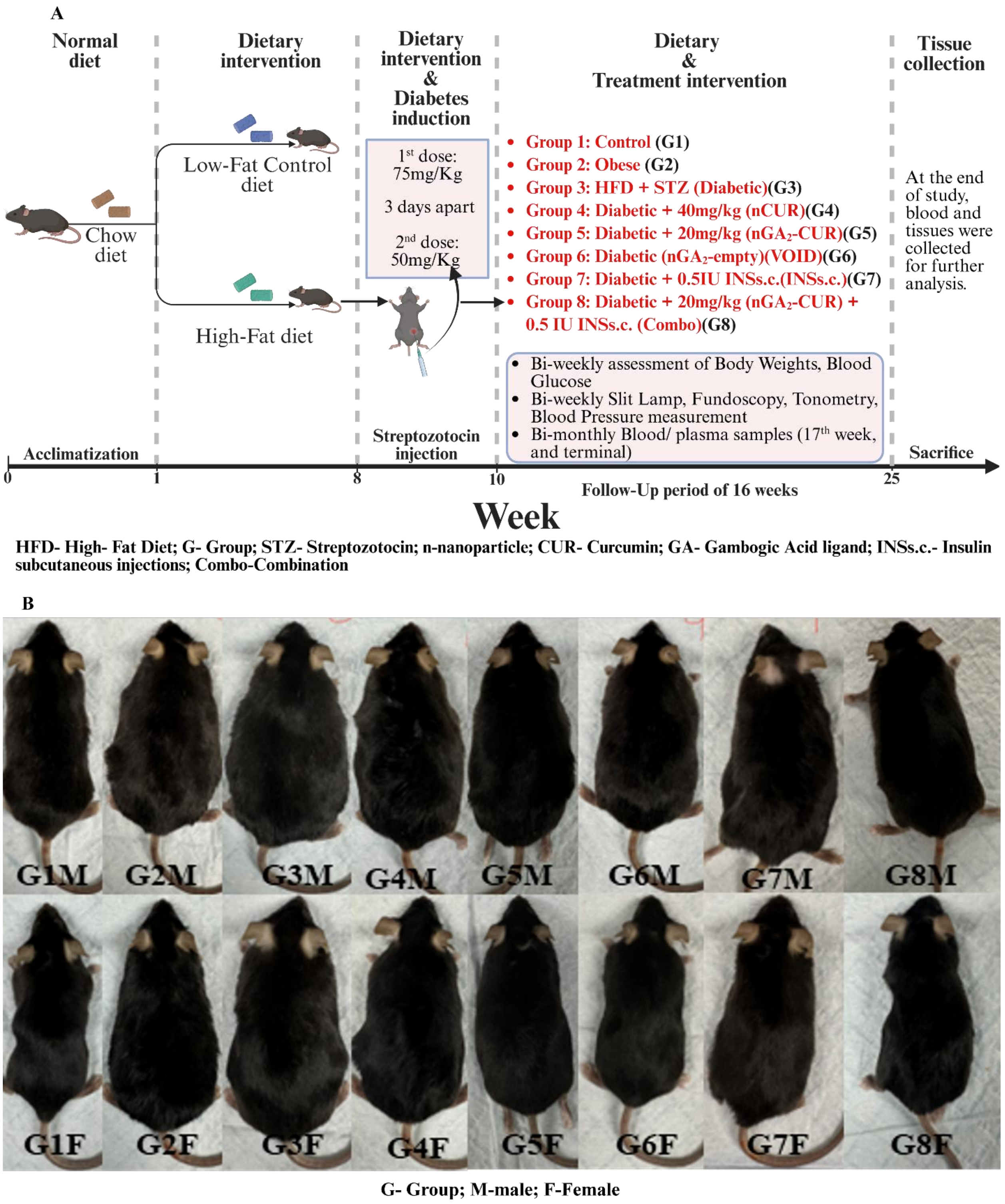

Figure S2: Study Plan, Related to STAR Methods. A) Shows the experimental plan of the study (Image created with Biorender), along with the representative images of male and female from each group (B).

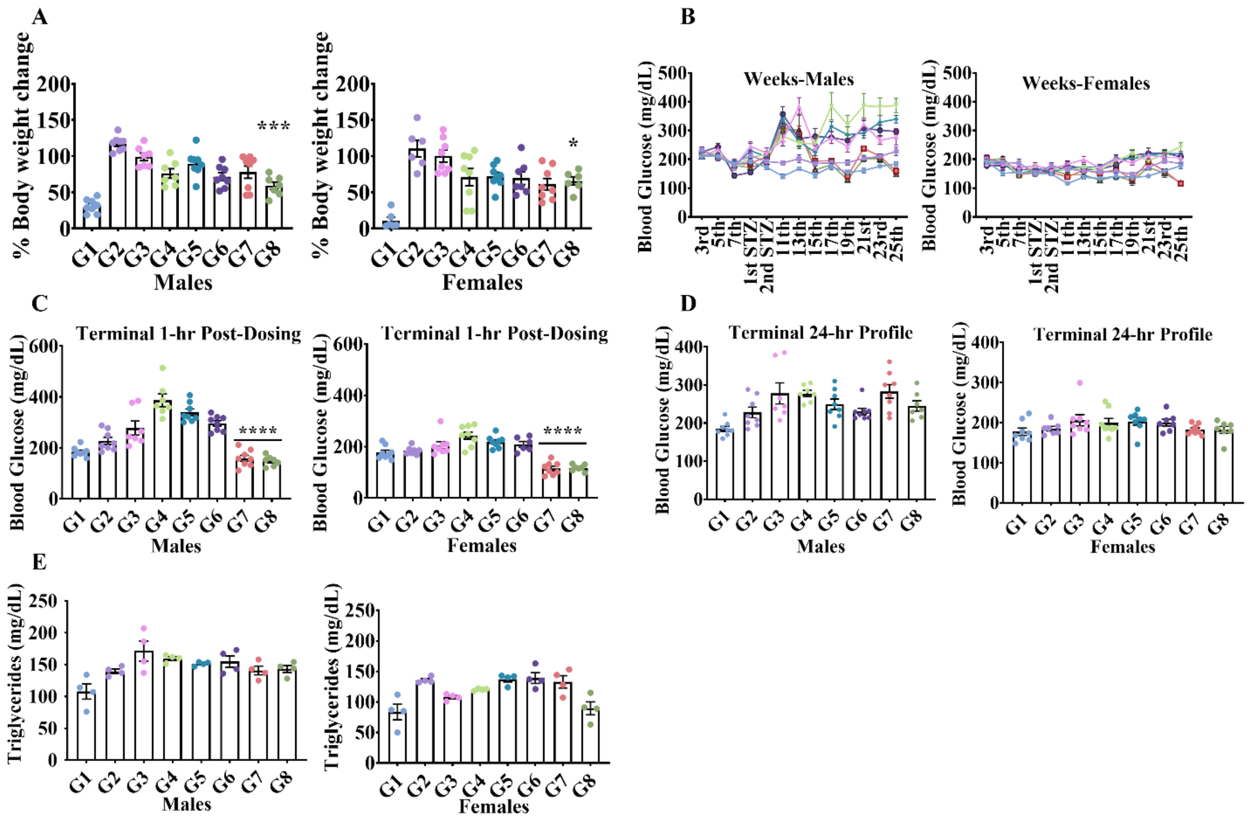

Figure S3: Systemic Metabolic Assessment: Body Weight, Glucose Homeostasis, and Lipid Profiles. Related to Figure 1. (A) Percentage change in body weights at the end of the study compared with initial weights. (B) Longitudinal blood glucose levels of both males and females over the 25-week study period. (C) Terminal blood glucose measured one-hour post-dosing. (D) 24-hour blood glucose profile measured in the terminal week of the study. (E) Terminal plasma triglyceride levels. Data are presented as mean ± SEM, with n=4-8 mice/ group. Longitudinal data (B) were assessed using a two-way ANOVA. All other datasets were analyzed using a one-way ANOVA followed by Dunnett's multiple comparison test. All comparisons were made relative to the Diabetic group (G3); for clarity, only the differences between the Diabetic group (G3) and Combination group (G8) are annotated. Significance levels: *p < 0.05, **p < 0.01, ***p < 0.001, and ****p < 0.0001.

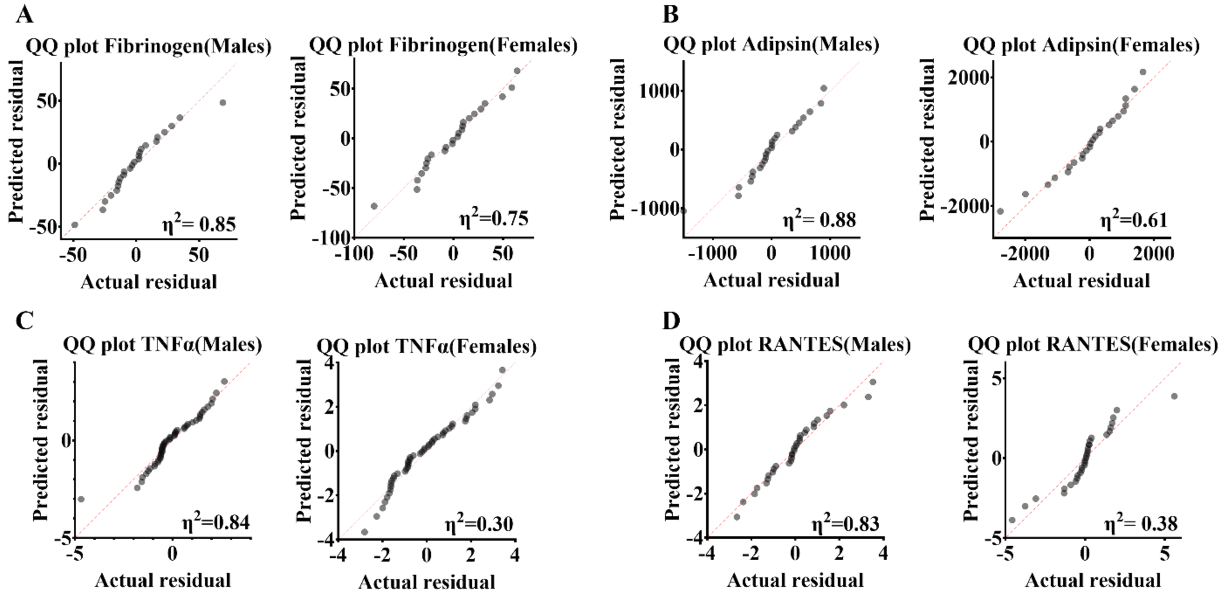

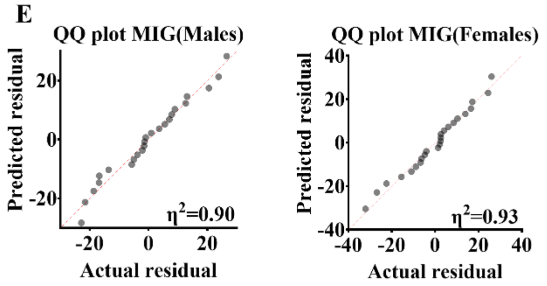

F

RANK -ORDER	G4: nCUR		G5:nGA$_2$-CUR		G6: VOID		G7: INSs.c.		G8: Combo	
RANKING	1- Most effective relative to Diabetic control<br>5- Least effective relative to Diabetic control									
Markers	Male	Female	Male	Female	Male	Female	Male	Female	Male	Female
Terminal Blood Glucose (mg/dL)	5	5	4	4	3	3	2	1	1	2
Fibrinogen (ng/mL)	1	3	5	4	4	5	2	1	3	2
Adipsin (ng/mL)	2	3	5	2	4	5	3	4	1	1
TNFα (pg/mL)	4	2	2	1	3	5	5	3	1	4
RANTES/CCL5 (pg/mL)	5	3	2	5	3	4	1	2	4	1
MIG/CXCL9 (pg/mL)	4	1	3	4	2	5	5	3	1	2
**Sum of ranking**	**21**	**17**	**21**	**20**	**19**	**27**	**18**	**14**	**11**	**12**
**Frequency of 1 (6 markers)**	**1**	**1**	**-**	**1**	**-**	**-**	**1**	**2**	**4**	**2**

Figure S4: Assessment of Data Normality and Efficacy Ranking for Markers Analyzed in Figure 1. (A-E) Quantile-Quantile (Q-Q) plots assessing the normality of data distribution for the key plasma markers shown in Figure 1 (male and female cohorts). The calculated effect size (η^2) is annotated within each panel. Markers include: (A) Plasma Fibrinogen, (B) Plasma Adipsin, (C) Plasma TNFα (D) Plasma RANTES (CCL5), and (E) Plasma MIG (CXCL9). (F) Treatment groups were rank-ordered relative to the Diabetic group (G3) for each analyzed marker. A rank of 1 indicates the most effective treatment for that specific marker, while a rank of 5 indicates the least effective. Overall therapeutic efficacy is determined by the lowest cumulative score and the highest frequency of top-tier rankings (Rank 1).

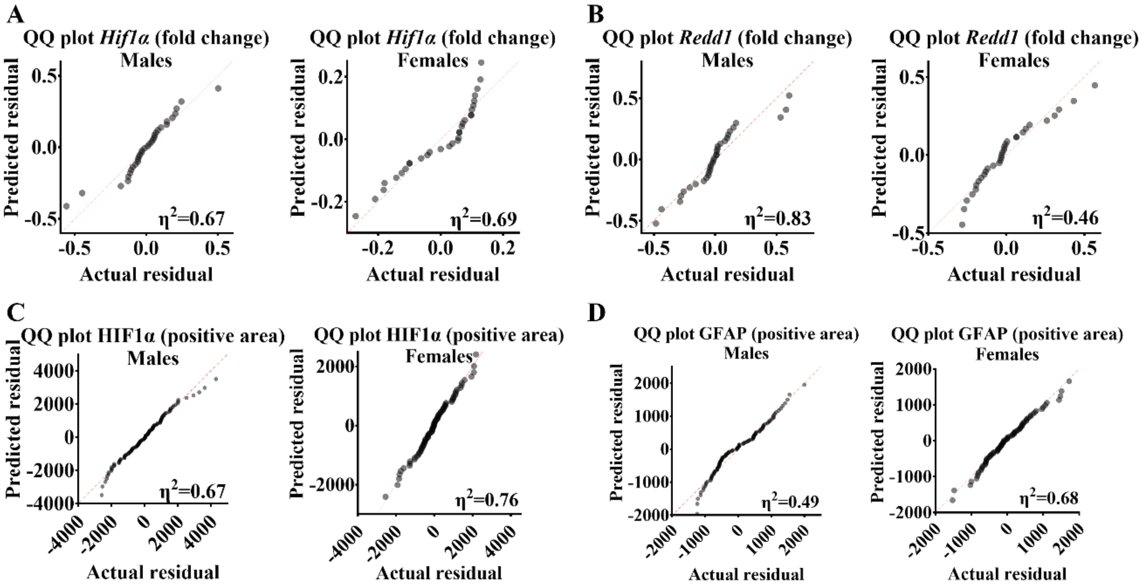

Figure S5: Assessment of Data Normality for Retinal Stress Markers Analyzed in Figure 2. (A-D) Quantile-Quantile (Q-Q) plots assessing the normality of data distribution for the retinal markers presented in Figure 2 (male and female cohorts). The calculated effect size (η^2) is annotated within each panel. Plots correspond to: (A-B) *Hif1α* and *Redd1* gene expression levels in retinal homogenates; (C) Quantification of HIF1α positive areas in retinal sections; and (D) Quantification of GFAP positive areas in retinal sections.

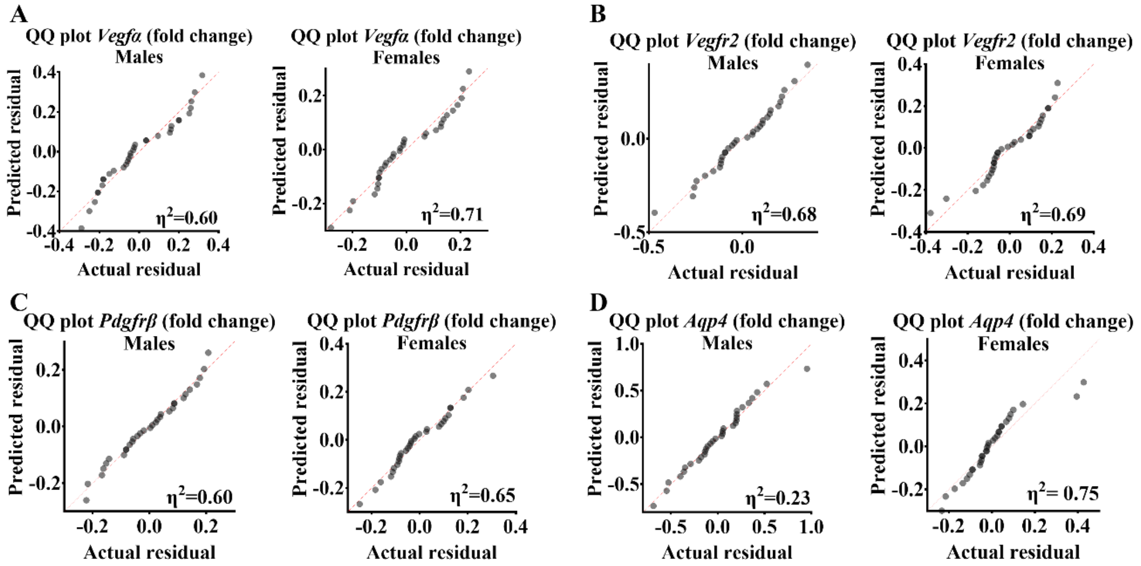

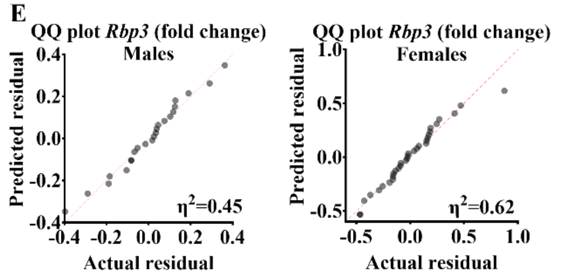

F

RANK -ORDER	G4: nCUR		G5: nGA_2-CUR		G6: VOID		G7: INSs.c.		G8: Combo	
RANKING	1- Most effective relative to Diabetic control<br>5- Least effective relative to Diabetic control									
Markers	Male	Female	Male	Female	Male	Female	Male	Female	Male	Female
HIF1α	1	2	3	3	2	4	4	5	5	1
REDD1	5	5	4	4	2	1	1	3	3	2
VEGFR2	2	2	1	3	3	4	5	5	4	1
VEGFA	1	2	3	3	4	5	5	4	2	1
PDGFRβ	3	2	1	5	5	4	4	3	2	1
AQP4	1	2	3	4	5	5	4	3	2	1
RBP3	1	2	4	3	3	5	5	4	2	1
Sum of ranking	14	17	19	25	24	28	28	27	20	8
Frequency of 1 (7 markers)	4	-	2	-	-	1	1	-	-	6

Figure S6: Assessment of Data Normality via Q-Q Plots and Cumulative Rank-Based Efficacy Analysis. Related to Figure 3. (A-E) Quantile-Quantile (Q-Q) plots assessing the normality of data distribution for the angiogenesis and fluid homeostasis markers presented in Figure 3 (male and female cohorts). The calculated effect size (η2) is annotated within each panel. Plots correspond to gene expression of: (A) *Vegfa*, (B) *Vegfr2*, (C) *Pdgfrβ*, (D) *Aqp4*, and (E) *Rbp3*. (F) Treatment groups were rank-ordered relative to the Diabetic group (G3) based on the combined data from Figure 2 and Figure 3. A rank of 1 indicates the most effective treatment for that specific marker, while a rank of 5 indicates the least effective. Overall therapeutic efficacy is determined by the lowest cumulative score and the highest frequency of top-tier rankings (Rank 1).

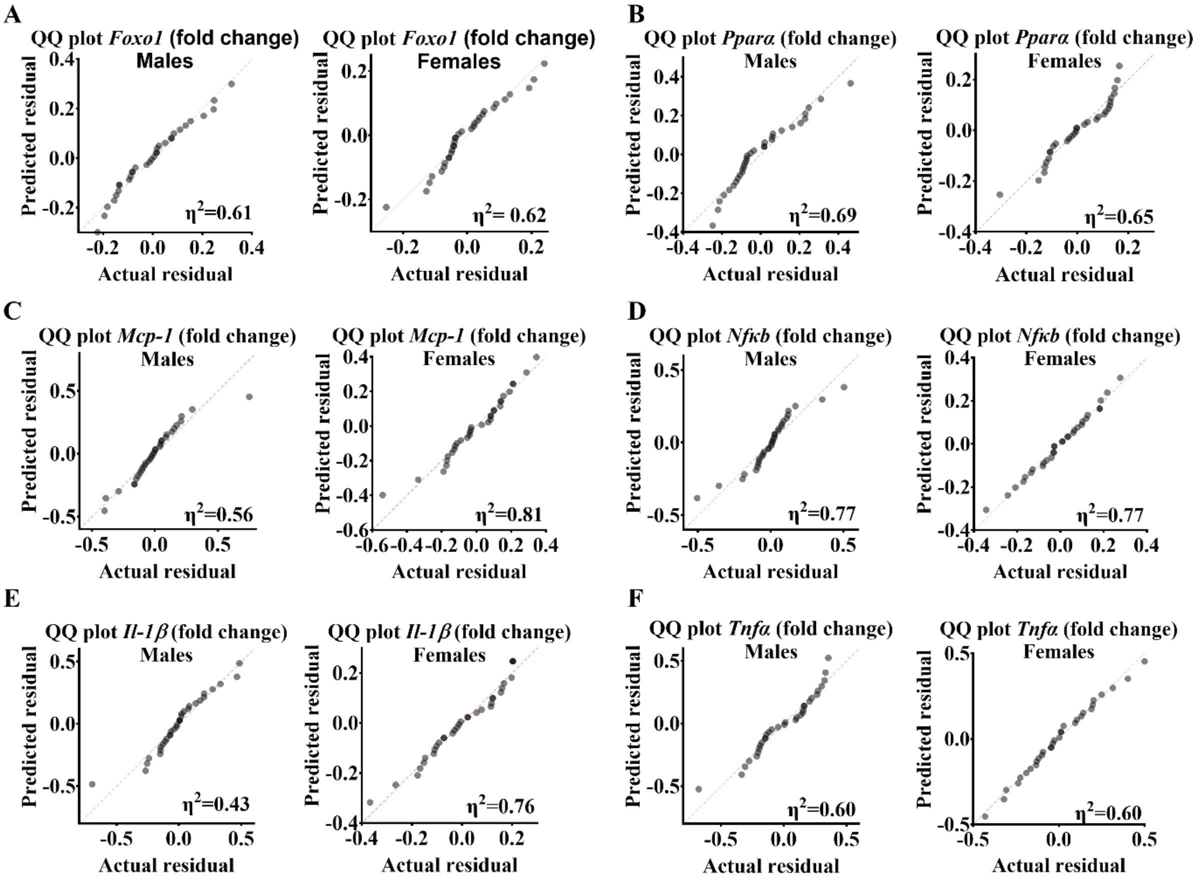

G

RANK -ORDER	G4: nCUR		G5: nGA_2-CUR		G6: VOID		G7: INSs.c.		G8: Combo	
RANKING	1- Most effective relative to Diabetic control<br>5- Least effective relative to Diabetic control									
Marker	Male	Female	Male	Female	Male	Female	Male	Female	Male	Female
FOXO1	1	2	4	4	3	3	1	5	5	1
PPARα	2	3	5	2	3	4	4	5	1	1
MCP1	2	2	4	4	5	5	1	3	3	1
NF-κB	1	1	4	4	2	5	5	3	3	2
IL-1β	2	2	4	3	5	5	3	4	1	1
TNFα	1	3	4	5	5	4	3	1	2	2
**Sum of ranking**	**8**	**12**	**21**	**18**	**21**	**21**	**12**	**18**	**12**	**6**
**Frequency of 1 (6 markers)**	**3**	**1**	**-**	**-**	**-**	**-**	**2**	**1**	**2**	**4**

Figure S7: Assessment of Data Normality via Q-Q Plots and Rank-Based Efficacy Analysis for Figure 4. (A-F) Quantile-Quantile (Q-Q) plots assessing the normality of data distribution for the transcription factors and inflammatory markers presented in Figure 4 (male and female cohorts). The calculated effect size (η^2) is annotated within each panel. Plots correspond to gene expression levels in retinal homogenates for: (A) *Foxo1*, (B) *Pparα*, (C) *Mcp-1*, (D) *Nfκb*, (E) *Il-1β*, and (F) *Tnfα*. (G) Treatment groups were rank-ordered relative to the Diabetic group (G3) for each analyzed marker. A rank of 1 indicates the most effective treatment for that specific marker, while a rank of 5 indicates the least effective. Overall therapeutic efficacy is determined by the lowest cumulative score and the highest frequency of top-tier rankings (Rank 1).

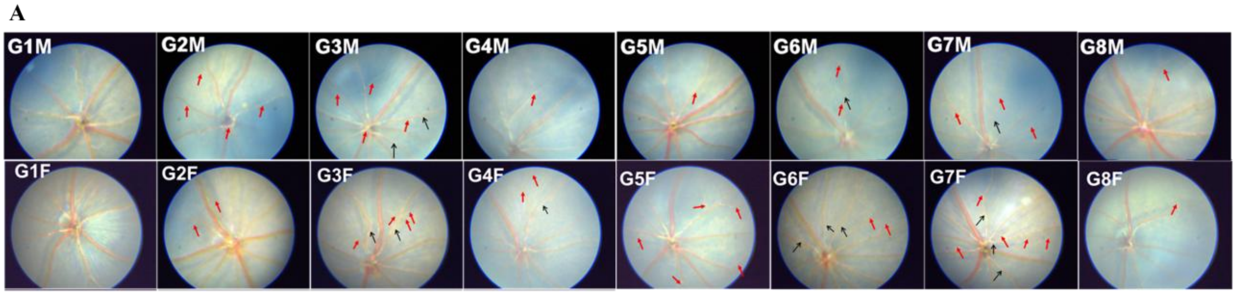

G1-Control; **G2**- Obese; **G3**- Diabetic; **G4**- nCUR; **G5**- nGA$_2$-CUR; **G6**-VOID; **G7**- INSs.c.; **G8-** Combo
G- Group; **M**-Male; **F**-Female

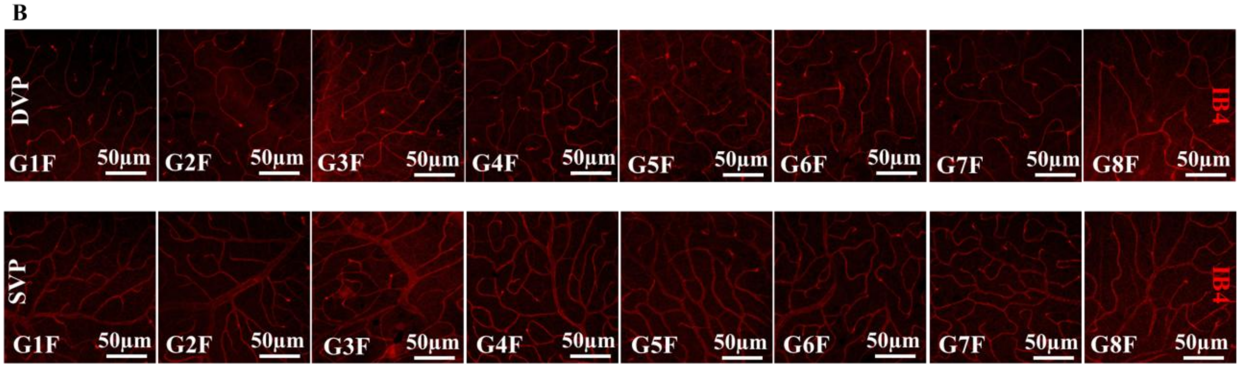

G- Group**; F-**Female

Figure S8: In Vivo Fundus Imaging and Ex Vivo Vascular Staining of Diabetic Retinopathy. Related to Figure 5. (A) Representative fundus photographs acquired at the 25-week study endpoint demonstrating signs of vascular remodeling. Red arrows indicate pathological angiogenesis, and the black arrow indicates vascular tortuosity. (B) Representative confocal images of female retinal whole mounts stained with Isolectin B4 (IB4) to visualize the vascular architecture of the Deep Vascular Plexus (DVP) and Superficial Vascular Plexus (SVP). Images were acquired at 20x magnification (scale bar = 50 µm).

A

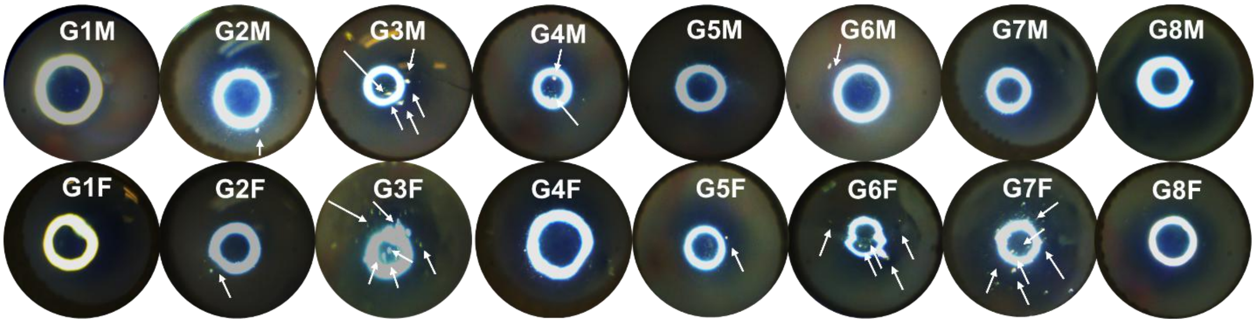

G1-Control; **G2**- Obese; **G3**- Diabetic; **G4**- nCUR; **G5**- nGA_2-CUR; **G6**-VOID; **G7**- INSs.c.; **G8**- Combo
G- Group; **M**-Male; **F**-Female

B

RANK -ORDER	G4: nCUR		G5: nGA_2-CUR		G6: VOID		G7: INSs.c.		G8: Combo	
RANKING	1- Most effective relative to Diabetic control									
	5- Least effective relative to Diabetic control									
Marker	Male	Female	Male	Female	Male	Female	Male	Female	Male	Female
Cataract Score	3	3	2	2	5	4	3	5	1	1
Intraocular Pressure (mmHg)	3	3	2	1	5	4	4	5	1	2
Sum of ranking	6	6	4	3	10	8	7	10	2	3
Frequency of 1 (2 markers)	-	-	-	1	-	-	-	-	2	1

Figure S9: Lens Transparency Evaluation and Overall Efficacy Analysis. Related to Figure 6. (A) Representative slit lamp images of lenses acquired at the 25-week study endpoint. White arrows indicate regions of lenticular opacity and punctate deposits characteristic of cataract formation in the Diabetic group (G3, both sexes) and Group G6. In contrast, the treatment groups (G5 & G8) exhibit clear lenses with transparency comparable to healthy controls (G1). (B) Treatment groups were rank-ordered relative to the Diabetic group (G3) based on the cataract and lens parameters analyzed in Figure 6. A rank of 1 indicates the most effective treatment for that specific marker, while a rank of 5 indicates the least effective. Overall therapeutic efficacy is determined by the lowest cumulative score and the highest frequency of top-tier rankings (Rank 1).

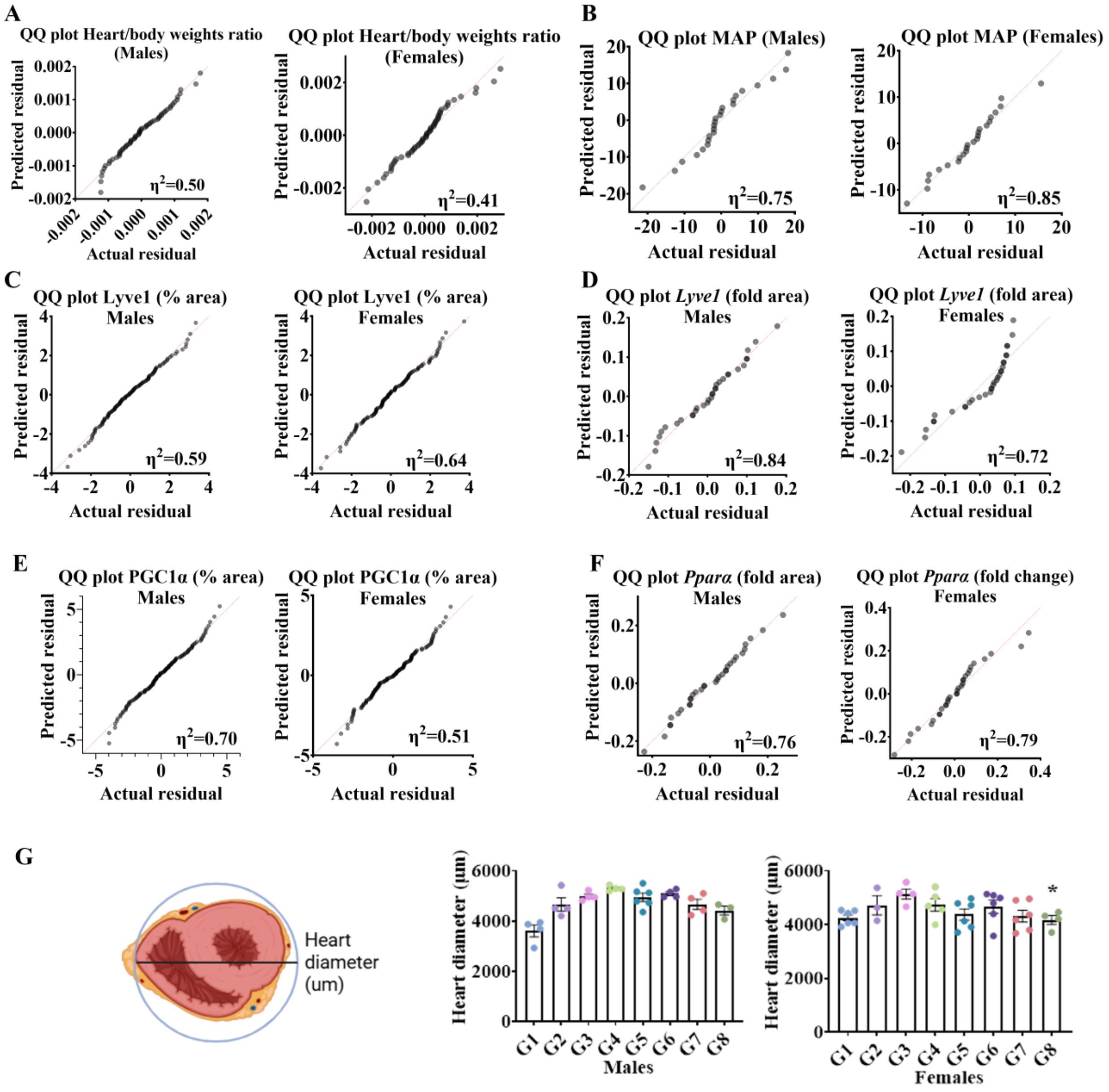

Figure S10: Assessment of Data Normality via Q-Q Plots for Cardiac Markers Analyzed in Figure 7. (A-F) Quantile-Quantile (Q-Q) plots assessing the normality of data distribution for the cardiac and metabolic markers presented in Figure 7 (male and female cohorts). Plots correspond to: (A) Heart-to-body weight ratios; (B) Mean Arterial Pressure (MAP) measured via tail-cuff method; (C) Quantification of LYVE1 positive area; (D) *Lyve1* gene expression in heart homogenates; (E) Quantification of PGC1α positive area; and (F) *Pparα* gene expression in heart homogenates. (G) Transverse heart diameter (µm) measured from histological cross-sections as a structural indicator of ventricular dilation. Representative schematic (left) illustrates the diameter measurement plane.

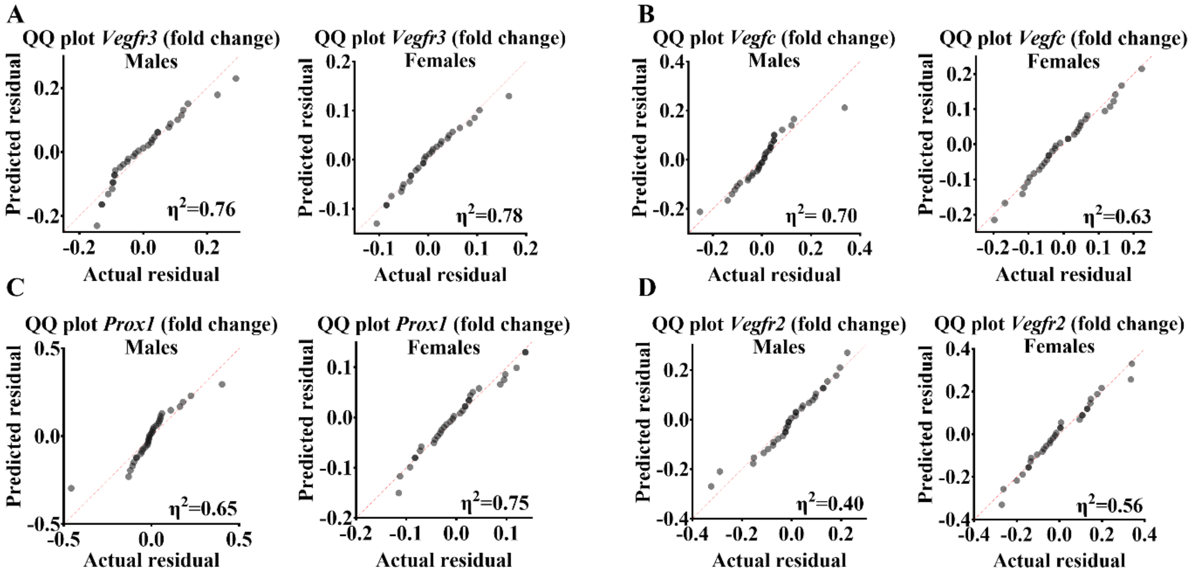

E

RANK -ORDER	G4: nCUR		G5: nGA$_2$-CUR		G6: VOID		G7: INSs.c.		G8: Combo	
RANKING	1- Most effective relative to Diabetic control<br>5- Least effective relative to Diabetic control									
Marker	Male	Female	Male	Female	Male	Female	Male	Female	Male	Female
LYVE1	4	4	1	1	5	5	3	3	2	2
VEGFR3	5	3	4	1	1	4	3	5	2	2
VEGFC	5	3	4	2	3	5	1	4	2	1
PROX1	4	2	3	3	5	5	1	4	2	1
VEGFR2	3	3	4	4	5	5	1	2	2	1
PPARα	5	4	3	2	4	5	2	3	1	1
Sum of ranking	**26**	**19**	**19**	**13**	**23**	**29**	**11**	**21**	**11**	**8**
Frequency of 1 (6 markers)	**-**	**-**	**1**	**2**	**1**	**-**	**3**	**-**	**1**	**4**

Figure S11: Assessment of Data Normality via Q-Q Plots and Rank-Based Efficacy Analysis for Figure 8. (A-D) Quantile-Quantile (Q-Q) plots assessing the normality of data distribution for the lymphangiogenic markers presented in Figure 8 (male and female cohorts). Plots correspond to gene expression levels in heart homogenates for: (A) *Vegfr3*, (B) *Vegfc*, (C) *Prox1*, and (D) *Vegfr2*. (E) Treatment groups were rank-ordered relative to the Diabetic group (G3) for each analyzed marker. A rank of 1 indicates the most effective treatment for that specific marker, while a rank of 5 indicates the least effective. Overall therapeutic efficacy is determined by the lowest cumulative score and the highest frequency of top-tier rankings (Rank 1).

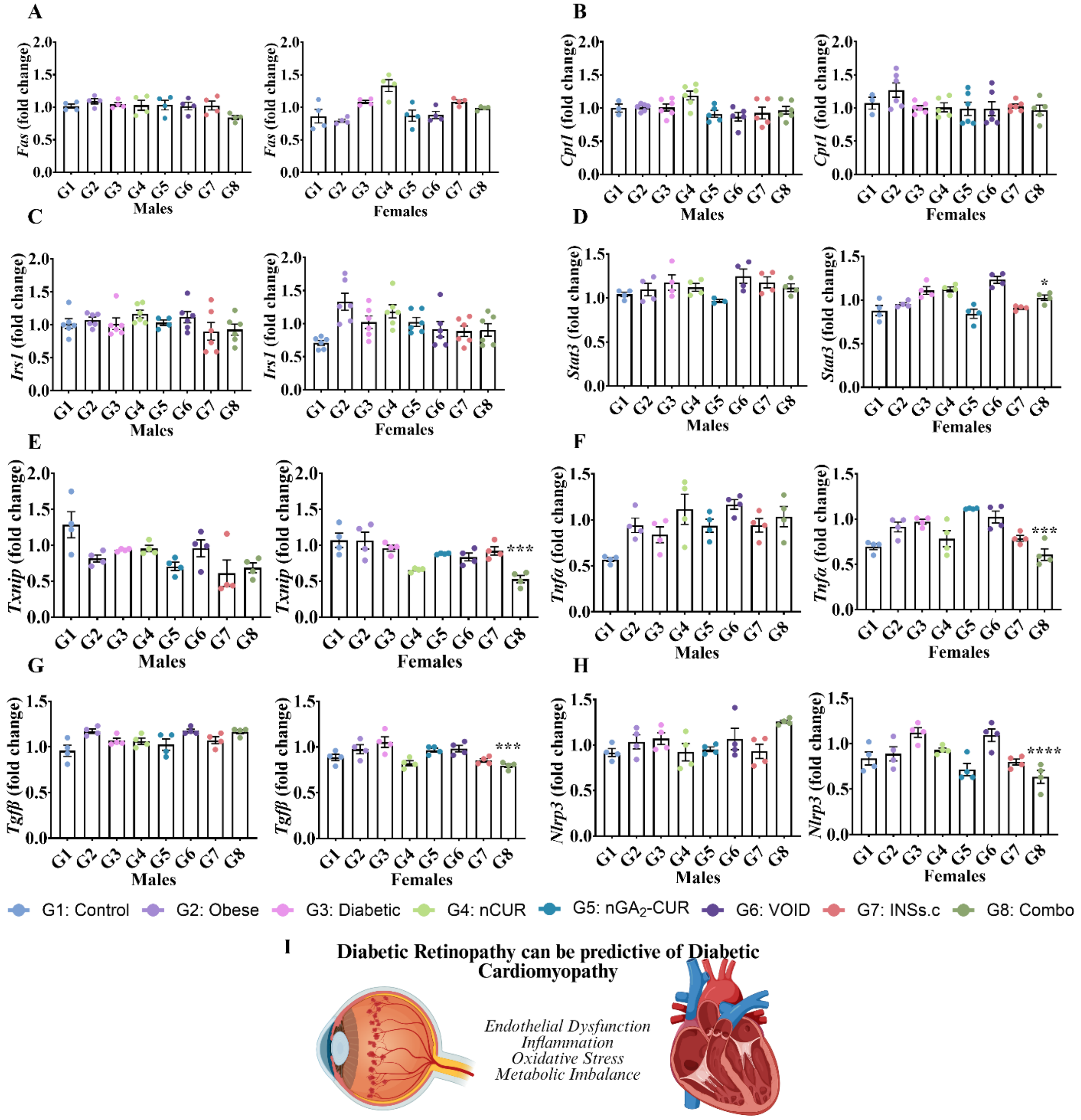

Figure S12: Transcriptional Profiling of Metabolic and Inflammatory Markers in Diabetic Cardiomyopathy. Related to Figure 8. (A-H) Real-time PCR analysis of mRNA levels for key metabolic and inflammatory markers in heart tissue: (A) *Fas*, (B) *Cpt1*, (C) *Irs1*, (D) *Stat3*, (E) *Txnip*, (F) *Tnfα*, (G) *Tgfβ*, and (H) *Nlrp3*. Data are normalized to the housekeeping gene β-actin and presented as fold change relative to controls. (I) Schematic diagram illustrates the proposed predictive relationship between the severity of Diabetic Retinopathy (DR) and the progression of Diabetic Cardiomyopathy (DCM). Data are presented as mean ± SEM. Data were analyzed using a one-way ANOVA followed by Dunnett's multiple comparison test (or Welch where appropriate) to determine significant differences relative to the Diabetic group (G3). Comparison annotations shown in the figure highlight differences between the Diabetic group (G3) and Combination group (G8). Significance levels: *$p < 0.05$, **$p < 0.01$, ***$p < 0.001$, and ****$p < 0.0001$.

Table S1: Animals used in the study

Group	Sex	Age (weeks)	Mice (n)	Duration of Study (weeks)	Dietary intervention	Diabetes induction (STZ injections)	Treatment	Untimely death or excluded from study
**Control (G1)**	Male	11	8	25	Low-Fat control	-	-	-
	Female	11	8	25	Low-Fat control	-	-	-
**Obese (G2)**	Male	11	8	25	High- Fat	-	-	-
	Female	11	8	25	High- Fat	-	-	1[#]
**Diabetic (G3)**	Male	11	8	25	High- Fat	2 doses	-	1[*]
	Female	11	8	25	High- Fat	2 doses	-	-
**nCUR (G4)**	Male	11	8	25	High- Fat	2 doses	Oral gavage of 40mg/kg equivalent CUR nanoparticle suspension	1[$]
	Female	11	8	25	High- Fat	2 doses		-
**nGA$_2$-CUR (G5)**	Male	11	8	25	High- Fat	2 doses	Oral gavage of 20mg/kg equivalent CUR nanoparticle suspension	-
	Female	11	8	25	High- Fat	2 doses		-
**VOID (G6)**	Male	11	8	25	High- Fat	2 doses	Oral gavage of empty nanoparticle suspension	-
	Female	11	8	25	High- Fat	2 doses		2[@$]
**INSs.c. (G7)**	Male	11	8	25	High- Fat	2 doses	Subcutaneous injection of Insulin glargine 0.5IU/kg	-
	Female	11	8	25	High- Fat	2 doses		-
**Combo (G8)**	Male	11	8	25	High- Fat	2 doses	Subcutaneous injection of Insulin glargine 0.5IU/kg followed by oral gavage of 20mg/kg equivalent CUR nanoparticle suspension	1[$]
	Female	11	8	25	High- Fat	2 doses		1[$]

[#] Died in the restrainer while monitoring blood pressure using CODA® High Throughput System.

[@]Mouse was severely injured due to aggressive encounters with cage mates and was subsequently excluded from the study; For other encounters, aggressive mice were separated and affected mice were treated as needed. Added more enrichment

[$] Mice did not survive the facial vein blood collection procedure during blood collections.

[*] Mouse died during fundoscopic examination

Table S2: Primer List for quantitative PCR

Gene	Forward (5'→3')	Reverse (5'→3')
Actb	GGCTGTATTCCCCTCCATCG	CCAGTTGGTAACAATGCCATGT
Hif1α	GATGACGGCGACATGGTTTAC	CTCACTGGGCCATTTCTGTGT
Vegfr2	CAAACCTCAATGTGTCTCTTTGC	AGAGTAAAGCCTATCTCGCTGT
Vegfa	GCACATAGAGAGAATGAGCTTCC	CTCCGCTCTGAACAAGGCT
Redd1	CTTCTGTGCGCCTTCATTCG	GTCAGGGACTGGCTGTAACC
Pdgfrβ	GACATTGAGTCCCCCAGCTAC	GATAGGTCCTTTCAGGGGCAGA
Rbp3	AAAGTGGGAGCTCAGCCATC	CCTGTTCCCACTGTCCTCAC
Aqp4	CTTTCTGGAAGGCAGTCTCAG	CCACACCGAGCAAAACAAAGAT
Foxo1	GGCGGGCTGGAAGAATTCAA	CCTCCCTCTGGATTGAGCATC
Pparα	TACTGCCGTTTTCACAAGTGC	AGGTCGTGTTCACAGGTAAGA
Mcp-1	CCACAACCACCTCAAGCACT	TAAGGCATCACAGTCCGAGTC
Tnfα	CCCTCACACTCAGATCATCTTCT	GCTACGACGTGGGCTACAG
Nfκb	ATGGCAGACGATGATCCCTAC	CGGAATCGAAATCCCCTCTGTT
IL-1β	GCAACTGTTCCTGAACTCAACT	ATCTTTTGGGGTCCGTCAACT
Irs1	CGATGGCTTCTCAGACGTG	CAGCCCGCTTGTTGATGTTG
Tgfβ	CCAAGGAGACGGAATACAGGG	GGGGCTGATCCCGTTGATT
Lyve1	CAGCACACTAGCCTGGTGTTA	CGCCCATGATTCTGCATGTAGA
Vegfr3	CTGGCAAATGGTTACTCCATGA	ACAACCCGTGTGTCTTCACTG
Vegfc	GAG GTC AAG GCT TTT GAA GGC	CTG TCC TGG TAT TGA GGG TGG
Prox1	AGA AGG GTTGACATTGGAGTGA	TGCGTGTTGCACCACAGAATA
Cpt1	TGGCATCATCACTGGTGTGTT	GTCTAGGGTCCGATTGATCTTTG
Fas	GCGGGTTCGTGAAACTGATAA	GCAAAATGGGCCTCCTTGATA
Stat3	AGAACCTCCAGGACGACTTTG	TCACAATGCTTCTCCGCATCT
Txnip	TCAATACCCCTGACCTAATGGC	TTCTGTCAATTCGAGCAGAGAC
Nlrp3	ATCAACAGGCGAGACCTCTG	GTCCTCCTGGCATACCATAGA

Table S3: Comprehensive Statistical Analysis of Blood glucose and Plasma Analytes. (Related to Figure 1)

Figure	Group comparisons	Summary	Adjusted p value
**1B- Males-Heat Map-Blood glucose levels**			
11th week	G3 vs. G1	****	<0.0001
	G3 vs. G2	*	0.015
	G3 vs. G5	*	0.0405
	G3 vs. G6	**	0.0042
13th week	G3 vs. G1	****	<0.0001
	G3 vs. G2	****	<0.0001
	G3 vs. G4	****	<0.0001
	G3 vs. G5	****	<0.0001
	G3 vs. G6	***	0.0001
	G3 vs. G7	**	0.0014
	G3 vs. G8	*	0.0118
15th week	G3 vs. G1	**	0.0038
	G3 vs. G8	*	0.031
17th week	G3 vs. G1	**	0.0013
	G3 vs. G2	**	0.0078
	G3 vs. G4	***	0.0004
	G3 vs. G7	*	0.0185
	G3 vs. G8	**	0.003
19th week	G3 vs. G1	**	0.0061
	G3 vs. G4	*	0.0316
	G3 vs. G7	**	0.0018
	G3 vs. G8	***	0.0006
21st week	G3 vs. G1	****	<0.0001
	G3 vs. G2	***	0.0001
	G3 vs. G7	*	0.0123
	G3 vs. G8	***	0.0002
23rd week	G3 vs. G1	**	0.0062
	G3 vs. G4	***	0.0002
25th week	G3 vs. G1	**	0.0038
	G3 vs. G4	***	0.0007
	G3 vs. G7	****	<0.0001
	G3 vs. G8	****	<0.0001
**1B- Females-Heat Map-Blood glucose levels**			
11th week	G3 vs. G1	****	<0.0001
	G3 vs. G7	**	0.0071
	G3 vs. G8	*	0.0389
13th week	G3 vs. G1	****	<0.0001
	G3 vs. G2	*	0.0223
	G3 vs. G7	*	0.0352
	G3 vs. G8	***	0.0006

15th week	G3 vs. G1	*	0.0352
	G3 vs. G8	**	0.0078
17th week	G3 vs. G1	****	<0.0001
	G3 vs. G2	**	0.0091
	G3 vs. G7	*	0.0391
	G3 vs. G8	****	<0.0001
19th week	G3 vs. G1	***	0.0007
	G3 vs. G2	*	0.0224
	G3 vs. G7	***	0.0005
	G3 vs. G8	***	0.0008
1D-Males-Fibrinogen	G1 vs. G3	*	0.0157
	G1 vs. G7	*	0.0217
	G2 vs. G3	*	0.0114
	G2 vs. G7	*	0.0298
	G3 vs. G4	****	<0.0001
	G3 vs. G5	***	0.0007
	G3 vs. G6	***	0.0006
	G3 vs. G7	****	<0.0001
	G3 vs. G8	****	<0.0001
1D-Females- Fibrinogen	G3 vs. G4	*	0.0108
	G3 vs. G7	**	0.0023
	G3 vs. G8	**	0.008
	G4 vs. G6	*	0.0244
	G6 vs. G7	**	0.0052
	G6 vs. G8	*	0.0182
1E-Males-Adipsin	G1 vs. G8	***	0.0004
	G2 vs. G4	**	0.0021
	G2 vs. G8	****	<0.0001
	G3 vs. G4	**	0.0056
	G3 vs. G8	****	<0.0001
	G4 vs. G8	*	0.022
	G5 vs. G8	****	<0.0001
	G6 vs. G8	***	0.0001
	G7 vs. G8	***	0.0004
1E-Females-Adipsin	G3 vs. G5	*	0.0496
	G3 vs. G7	*	0.0234
	G3 vs. G8	*	0.0422
1F-Males-TNFα	G1 vs. G2	****	<0.0001
	G1 vs. G3	****	<0.0001
	G2 vs. G4	**	0.0086
	G2 vs. G5	****	<0.0001
	G2 vs. G6	****	<0.0001
	G2 vs. G7	*	0.0151
	G2 vs. G8	****	<0.0001
	G3 vs. G4	***	0.0006
	G3 vs. G5	****	<0.0001

	G3 vs. G6	****	<0.0001
	G3 vs. G7	**	0.0011
	G3 vs. G8	****	<0.0001
	G4 vs. G5	**	0.0013
	G4 vs. G6	**	0.003
	G4 vs. G8	****	<0.0001
	G5 vs. G7	***	0.0007
	G6 vs. G7	**	0.0016
	G7 vs. G8	****	<0.0001
1F-Females-TNFα	G3 vs. G5	*	0.036
1G-Males-RANTES	G1 vs. G3	****	<0.0001
	G2 vs. G3	****	<0.0001
	G3 vs. G4	***	0.0002
	G3 vs. G5	****	<0.0001
	G3 vs. G6	****	<0.0001
	G3 vs. G7	****	<0.0001
	G3 vs. G8	****	<0.0001
	G4 vs. G7	*	0.0498
1G-Females-RANTES	G5 vs. G8	*	0.0439
1H-Males-MIG/CXCL9	G1 vs. G3	****	<0.0001
	G1 vs. G7	*	0.0161
	G2 vs. G3	***	0.0008
	G2 vs. G8	**	0.003
	G3 vs. G4	****	<0.0001
	G3 vs. G5	****	<0.0001
	G3 vs. G6	****	<0.0001
	G3 vs. G7	**	0.0062
	G3 vs. G8	****	<0.0001
	G7 vs. G8	***	0.0004
1H-Females-MIG/CXCL9	G1 vs. G4	*	0.0251
	G1 vs. G6	****	<0.0001
	G1 vs. G8	*	0.0353
	G2 vs. G3	*	0.0495
	G2 vs. G6	****	<0.0001
	G3 vs. G4	**	0.0021
	G3 vs. G6	***	0.0002
	G3 vs. G7	*	0.0309
	G3 vs. G8	**	0.003
	G4 vs. G6	****	<0.0001
	G5 vs. G6	****	<0.0001
	G6 vs. G7	****	<0.0001
	G6 vs. G8	****	<0.0001

Table S4*:* Statistical Evaluation of Cellular Stress Markers. (Related to Figure 2)

Figure	Group comparisons	Summary	Adjusted p value
2A-Males- *Hif1α*	G1 vs. G4	*	0.0343
	G1 vs. G5	**	0.0014
	G1 vs. G6	**	0.0039
	G1 vs. G7	***	0.0007
	G1 vs. G8	***	0.0004
	G2 vs. G8	*	0.0343
2A-Females-*Hif1α*	G1 vs. G3	**	0.003
	G1 vs. G6	**	0.0013
	G1 vs. G7	***	0.0004
	G3 vs. G8	*	0.0174
	G6 vs. G8	**	0.0076
	G7 vs. G8	**	0.0027
2B-Males-*Redd1*	G1 vs. G3	*	0.0357
	G1 vs. G4	****	<0.0001
	G1 vs. G5	**	0.0056
	G2 vs. G4	****	<0.0001
	G2 vs. G5	*	0.0153
	G3 vs. G4	***	0.0008
	G4 vs. G5	**	0.0053
	G4 vs. G6	****	<0.0001
	G4 vs. G7	****	<0.0001
	G4 vs. G8	****	<0.0001
	G5 vs. G7	*	0.0367
2E-Males- HIF1α-IF	G1 vs. G2	****	<0.0001
	G1 vs. G4	***	0.0003
	G1 vs. G5	****	<0.0001
	G1 vs. G6	****	<0.0001
	G1 vs. G7	****	<0.0001
	G1 vs. G8	****	<0.0001
	G2 vs. G3	****	<0.0001
	G2 vs. G6	*	0.0315
	G2 vs. G8	*	0.0124
	G3 vs. G5	****	<0.0001
	G3 vs. G6	****	<0.0001
	G3 vs. G7	****	<0.0001
	G3 vs. G8	****	<0.0001
	G4 vs. G6	****	<0.0001
	G4 vs. G7	****	<0.0001
	G4 vs. G8	****	<0.0001
2E-Females- HIF1α-IF	G1 vs. G2	****	<0.0001
	G1 vs. G3	****	<0.0001

	G1 vs. G4	****	<0.0001
	G1 vs. G5	****	<0.0001
	G1 vs. G6	****	<0.0001
	G1 vs. G7	****	<0.0001
	G1 vs. G8	****	<0.0001
	G2 vs. G3	****	<0.0001
	G2 vs. G4	**	0.003
	G2 vs. G6	*	0.0486
	G2 vs. G7	*	0.0156
	G3 vs. G5	****	<0.0001
	G3 vs. G6	*	0.0147
	G3 vs. G8	****	<0.0001
	G4 vs. G5	*	0.0471
	G4 vs. G8	****	<0.0001
	G6 vs. G8	**	0.0022
	G7 vs. G8	***	0.0008
2F-Males-GFAP-IF	G1 vs. G6	****	<0.0001
	G1 vs. G8	***	0.0002
	G2 vs. G3	***	0.0006
	G2 vs. G6	**	0.0033
	G3 vs. G4	**	0.0018
	G3 vs. G5	****	<0.0001
	G3 vs. G6	****	<0.0001
	G3 vs. G7	****	<0.0001
	G3 vs. G8	****	<0.0001
	G4 vs. G6	***	0.0001
	G4 vs. G8	*	0.0192
	G6 vs. G7	**	0.0063
2F-Females-GFAP-IF	G1 vs. G2	****	<0.0001
	G1 vs. G3	****	<0.0001
	G1 vs. G4	****	<0.0001
	G1 vs. G5	****	<0.0001
	G1 vs. G6	****	<0.0001
	G1 vs. G7	***	0.0001
	G1 vs. G8	****	<0.0001
	G2 vs. G3	****	<0.0001
	G2 vs. G4	*	0.0435
	G2 vs. G6	****	<0.0001
	G3 vs. G7	***	0.0008
	G3 vs. G8	**	0.0027
	G6 vs. G7	***	0.0005
	G6 vs. G8	**	0.0019

Table S5: Statistical Evaluation of Angiogenesis and Fluid Homeostasis Markers. (Related to Figure 3)

Figure	Group comparisons	Summary	Adjusted p value
3A-Males-*Vegfa*	G1 vs. G3	**	0.0025
	G1 vs. G7	*	0.0149
	G2 vs. G3	*	0.0149
	G3 vs. G4	*	0.0155
3A-Females- *Vegfa*	G1 vs. G3	**	0.0012
	G1 vs. G6	*	0.0181
	G1 vs. G7	*	0.0191
	G2 vs. G3	*	0.0276
	G3 vs. G8	****	<0.0001
	G4 vs. G8	*	0.0416
	G5 vs. G8	*	0.0276
	G6 vs. G8	**	0.0011
	G7 vs. G8	**	0.0012
3B-Males- *Vegfr2*	G1 vs. G7	***	0.0006
	G2 vs. G7	**	0.0023
	G3 vs. G5	*	0.0171
	G4 vs. G7	**	0.0058
	G5 vs. G7	***	0.0001
3B-Females- *Vegfr2*	G1 vs. G3	*	0.0284
	G1 vs. G7	*	0.0345
	G2 vs. G8	*	0.0245
	G3 vs. G8	***	0.0001
	G6 vs. G8	**	0.0022
	G7 vs. G8	***	0.0001
3C-Males-*Pdgfrβ*	G1 vs. G3	*	0.0122
	G2 vs. G3	*	0.0373
	G3 vs. G5	**	0.0043
	G3 vs. G8	**	0.0063
	G5 vs. G6	*	0.0395
3C-Females- *Pdgfrβ*	G1 vs. G3	*	0.0231
	G2 vs. G8	*	0.0289
	G3 vs. G4	*	0.0122
	G3 vs. G7	*	0.0173
	G3 vs. G8	****	<0.0001
	G5 vs. G8	*	0.0163
	G6 vs. G8	*	0.0273
3D-Females- *Aqp4*	G1 vs. G5	*	0.0182
	G1 vs. G6	***	0.0002
	G2 vs. G6	**	0.0071
	G3 vs. G8	**	0.006

	G4 vs. G6	**	0.0031
	G5 vs. G8	***	0.0004
	G6 vs. G7	**	0.0054
	G6 vs. G8	****	<0.0001
3E-Females- *Rbp3*	G1 vs. G6	*	0.0191
	G1 vs. G7	*	0.0345
	G2 vs. G3	*	0.0121
	G2 vs. G5	*	0.0201
	G2 vs. G6	**	0.0027
	G2 vs. G7	**	0.0052

Table S6: Statistical Evaluation of Transcription Factors and Chronic Inflammatory Markers. (Related to Figure 4)

Figure	Group comparisons	Summary	Adjusted p value
4A-Males-*Foxo1*	G1 vs. G3	**	0.0014
	G1 vs. G4	*	0.0389
	G1 vs. G5	**	0.0048
	G1 vs. G6	**	0.0087
	G1 vs. G7	*	0.0389
	G1 vs. G8	***	0.0005
4A-Females- *Foxo1*	G2 vs. G8	**	0.003
	G3 vs. G8	**	0.0044
	G7 vs. G8	***	0.0003
4B-Males-*Pparα*	G1 vs. G8	**	0.0022
	G2 vs. G4	*	0.0215
	G2 vs. G8	***	0.0002
	G3 vs. G8	*	0.0119
	G4 vs. G5	*	0.0224
	G5 vs. G8	***	0.0002
	G7 vs. G8	**	0.0014
4B-Females- *Pparα*	G1 vs. G6	**	0.0039
	G1 vs. G7	**	0.0016
	G2 vs. G8	*	0.0484
	G6 vs. G8	**	0.0031
	G7 vs. G8	**	0.0012
4C-Males-*Mcp*-1	G1 vs. G6	**	0.0017
	G2 vs. G6	*	0.0128
	G6 vs. G7	*	0.0152
4C-Females- *Mcp-1*	G1 vs. G3	****	<0.0001
	G1 vs. G5	**	0.0043
	G1 vs. G6	****	<0.0001
	G1 vs. G7	**	0.0046
	G2 vs. G6	**	0.0021
	G3 vs. G8	***	0.0001
	G4 vs. G6	**	0.0012
	G5 vs. G6	*	0.0495
	G5 vs. G8	**	0.0061
	G6 vs. G7	*	0.0461
	G6 vs. G8	****	<0.0001
	G7 vs. G8	**	0.0067
4D-Males-*Nfκb*	G1 vs. G5	****	<0.0001
	G1 vs. G6	*	0.0159
	G1 vs. G7	****	<0.0001
	G1 vs. G8	**	0.0029

	G2 vs. G5	***	0.0003
	G2 vs. G7	****	<0.0001
	G2 vs. G8	*	0.0105
	G3 vs. G5	*	0.0461
	G3 vs. G7	**	0.0072
	G4 vs. G5	*	0.0278
	G4 vs. G7	**	0.0042
4D-Females- *Nfκb*	G1 vs. G3	*	0.0133
	G1 vs. G6	****	<0.0001
	G2 vs. G6	***	0.0001
	G3 vs. G4	**	0.0058
	G3 vs. G8	*	0.0155
	G4 vs. G6	****	<0.0001
	G5 vs. G6	*	0.0232
	G6 vs. G7	**	0.0012
	G6 vs. G8	****	<0.0001
4E-Males-*Il-1β*	G1 vs. G3	*	0.0443
4E-Females- *Il-1β*	G1 vs. G3	***	0.0009
	G1 vs. G6	***	0.0003
	G2 vs. G3	*	0.03
	G2 vs. G6	**	0.0093
	G3 vs. G8	****	<0.0001
	G4 vs. G6	*	0.0416
	G4 vs. G8	*	0.0345
	G5 vs. G8	*	0.0205
	G6 vs. G8	****	<0.0001
	G7 vs. G8	*	0.0119
4F-Males-*Tnfα*	G1 vs. G3	**	0.0045
	G1 vs. G6	*	0.043
	G2 vs. G3	*	0.0198
	G3 vs. G4	**	0.0076
	G3 vs. G8	*	0.0306
4F-Females- *Tnfα*	G3 vs. G7	**	0.0032
	G3 vs. G8	**	0.0084
	G5 vs. G7	*	0.0111
	G5 vs. G8	*	0.0278

Table S7: Statistical Evaluation of Retinal Vascular Topology and Glial Activation (Female Cohort). (Related to Figure 5)

Figure	Group comparisons	Summary	Adjusted p value
5B-DVP-Total # Junctions	G1 vs. G3	***	0.0004
	G2 vs. G3	***	0.0002
	G3 vs. G5	***	0.0002
	G3 vs. G6	****	<0.0001
	G3 vs. G7	****	<0.0001
	G3 vs. G8	****	<0.0001
	G4 vs. G6	*	0.0347
	G4 vs. G7	*	0.0452
5C-DVP-Total vessel length	G2 vs. G3	*	0.0156
	G2 vs. G4	**	0.0038
	G3 vs. G5	**	0.0023
	G3 vs. G6	***	0.0002
	G3 vs. G7	***	0.0007
	G3 vs. G8	***	0.0008
	G4 vs. G5	***	0.0006
	G4 vs. G6	****	<0.0001
	G4 vs. G7	***	0.0001
	G4 vs. G8	***	0.0001
5D-DVP-Mean Lacuranity	G2 vs. G3	*	0.045
	G2 vs. G4	*	0.0303
	G3 vs. G6	**	0.0042
	G3 vs. G7	*	0.0117
	G3 vs. G8	*	0.0128
	G4 vs. G6	**	0.0027
	G4 vs. G7	**	0.0078
	G4 vs. G8	**	0.0084
5E-DVP- Vessel (%) area	G1 vs. G4	**	0.0095
	G2 vs. G4	**	0.0016
	G3 vs. G6	*	0.027
	G3 vs. G7	*	0.0475
	G3 vs. G8	*	0.0427
	G4 vs. G5	*	0.0188
	G4 vs. G6	***	0.0006
	G4 vs. G7	**	0.0012
	G4 vs. G8	**	0.001
5G-SVP-Total # Junctions	G3 vs. G4	**	0.0032
	G3 vs. G5	*	0.0117

	G3 vs. G6	**	0.0014
	G3 vs. G7	**	0.0033
	G3 vs. G8	****	<0.0001
5H-SVP-Total vessel length (mm)	G1 vs. G8	**	0.003
	G2 vs. G8	**	0.0011
	G3 vs. G5	***	0.0003
	G3 vs. G6	***	0.0004
	G3 vs. G7	***	0.0003
	G3 vs. G8	****	<0.0001
5I-SVP-Mean Lacuranity	G1 vs. G3	**	0.0017
	G1 vs. G7	**	0.0022
	G3 vs. G6	**	0.0017
	G3 vs. G8	**	0.0062
	G6 vs. G7	**	0.0017
	G7 vs. G8	**	0.0058
5J-SVP- Vessel (%) area	G1 vs. G3	**	0.0064
	G1 vs. G7	*	0.0164
	G3 vs. G8	*	0.0166
	G7 vs. G8	*	0.033
5M-Flat mount- IBA1 (% area)	G1 vs. G3	**	0.0076
	G1 vs. G4	**	0.004
	G1 vs. G6	****	<0.0001
	G1 vs. G7	****	<0.0001
	G2 vs. G6	****	<0.0001
	G2 vs. G7	****	<0.0001
	G3 vs. G5	**	0.0031
	G3 vs. G8	*	0.0362
	G4 vs. G5	**	0.0018
	G4 vs. G6	*	0.0176
	G4 vs. G7	*	0.0175
	G4 vs. G8	*	0.0237
	G5 vs. G6	****	<0.0001
	G5 vs. G7	****	<0.0001
	G6 vs. G8	****	<0.0001
	G7 vs. G8	****	<0.0001
5N- Flat mount-GFAP (% area)	G1 vs. G2	*	0.0422
	G1 vs. G3	***	0.0007
	G1 vs. G4	****	<0.0001
	G1 vs. G6	***	0.0004
	G1 vs. G7	****	<0.0001

	G1 vs. G8	****	<0.0001
	G2 vs. G4	**	0.0041
	G2 vs. G5	**	0.0015
	G2 vs. G7	***	0.0006
	G3 vs. G5	****	<0.0001
	G4 vs. G5	****	<0.0001
	G5 vs. G6	****	<0.0001
	G5 vs. G7	****	<0.0001
	G5 vs. G8	****	<0.0001
	G6 vs. G7	*	0.0386

Table S8: Statistical Evaluation of Cataract Progression and Lens Biophysical Properties. (Related to Figure 6)

Figure	Group comparisons	Summary	Adjusted p value
6A-Males-Cataract scores	G1 vs. G2	*	0.0452
	G1 vs. G3	****	<0.0001
	G1 vs. G4	****	<0.0001
	G1 vs. G5	****	<0.0001
	G1 vs. G6	****	<0.0001
	G1 vs. G7	****	<0.0001
	G1 vs. G8	**	0.0027
	G2 vs. G3	****	<0.0001
	G2 vs. G4	****	<0.0001
	G2 vs. G5	****	<0.0001
	G2 vs. G6	****	<0.0001
	G2 vs. G7	****	<0.0001
	G3 vs. G5	****	<0.0001
	G3 vs. G6	**	0.008
	G3 vs. G8	****	<0.0001
	G4 vs. G5	****	<0.0001
	G4 vs. G6	**	0.008
	G4 vs. G8	****	<0.0001
	G5 vs. G6	****	<0.0001
	G5 vs. G7	****	<0.0001
	G5 vs. G8	****	<0.0001
	G6 vs. G7	**	0.008
	G6 vs. G8	****	<0.0001
	G7 vs. G8	****	<0.0001
6A-Females-Cataract scores	G1 vs. G2	****	<0.0001
	G1 vs. G3	****	<0.0001
	G1 vs. G4	****	<0.0001
	G1 vs. G5	**	0.0021
	G1 vs. G6	****	<0.0001
	G1 vs. G7	****	<0.0001
	G2 vs. G3	****	<0.0001
	G2 vs. G4	****	<0.0001
	G2 vs. G5	***	0.0002
	G2 vs. G6	****	<0.0001
	G2 vs. G7	****	<0.0001
	G2 vs. G8	****	<0.0001
	G3 vs. G4	*	0.0111
	G3 vs. G5	****	<0.0001
	G3 vs. G8	****	<0.0001

	G4 vs. G5	****	<0.0001
	G4 vs. G7	****	<0.0001
	G4 vs. G8	****	<0.0001
	G5 vs. G6	****	<0.0001
	G5 vs. G7	****	<0.0001
	G6 vs. G8	****	<0.0001
	G7 vs. G8	****	<0.0001
6B-Males-IOP	G1 vs. G2	***	0.0002
	G1 vs. G3	****	<0.0001
	G1 vs. G4	****	<0.0001
	G1 vs. G5	**	0.0022
	G1 vs. G6	****	<0.0001
	G1 vs. G7	****	<0.0001
	G1 vs. G8	*	0.0142
	G2 vs. G6	****	<0.0001
	G2 vs. G7	*	0.038
	G3 vs. G5	**	0.01
	G3 vs. G6	***	0.0009
	G3 vs. G8	*	0.0234
	G4 vs. G6	****	<0.0001
	G5 vs. G6	****	<0.0001
	G5 vs. G7	**	0.0052
	G6 vs. G7	***	0.0003
	G6 vs. G8	****	<0.0001
	G7 vs. G8	*	0.0155
6B-Females-IOP	G1 vs. G2	****	<0.0001
	G1 vs. G3	****	<0.0001
	G1 vs. G4	***	0.0003
	G1 vs. G6	****	<0.0001
	G1 vs. G7	****	<0.0001
	G1 vs. G8	*	0.0482
	G2 vs. G6	**	0.0023
	G2 vs. G7	****	<0.0001
	G3 vs. G4	**	0.001
	G3 vs. G5	****	<0.0001
	G3 vs. G7	*	0.0148
	G3 vs. G8	****	<0.0001
	G4 vs. G6	****	<0.0001
	G4 vs. G7	****	<0.0001
	G5 vs. G6	****	<0.0001
	G5 vs. G7	****	<0.0001
	G6 vs. G8	****	<0.0001
	G7 vs. G8	****	<0.0001

Table S10: Statistical Evaluation of Early Lymphangiogenic Markers. (Related to Figure 8)

Figure	Group comparisons	Summary	Adjusted p value
8A- Males-*Vegfr3*	G1 vs. G6	**	0.0031
	G2 vs. G5	*	0.0407
	G2 vs. G6	****	<0.0001
	G2 vs. G7	**	0.0076
	G2 vs. G8	***	0.0007
	G3 vs. G6	****	<0.0001
	G3 vs. G7	*	0.0294
	G3 vs. G8	**	0.0029
	G4 vs. G6	***	0.0004
	G4 vs. G8	*	0.0132
8A-Females-*Vegfr3*	G1 vs. G3	**	0.0053
	G1 vs. G5	*	0.0403
	G2 vs. G3	**	0.0046
	G2 vs. G5	*	0.0452
	G3 vs. G4	****	<0.0001
	G3 vs. G5	****	<0.0001
	G3 vs. G6	***	0.0001
	G3 vs. G7	***	0.0008
	G3 vs. G8	****	<0.0001
8B-Males-*Vegfc*	G2 vs. G5	**	0.0075
	G2 vs. G6	**	0.0064
	G2 vs. G7	***	0.0005
	G2 vs. G8	**	0.0012
	G3 vs. G7	*	0.0128
	G3 vs. G8	*	0.0269
	G4 vs. G5	*	0.0186
	G4 vs. G6	*	0.016
	G4 vs. G7	**	0.0014
	G4 vs. G8	**	0.0032
8B-Females-*Vegfc*	G1 vs. G3	*	0.0254
	G2 vs. G8	*	0.0107
	G3 vs. G5	**	0.0041
	G3 vs. G8	***	0.0002
8C- Males- *Prox1*	G2 vs. G7	**	0.0088
	G2 vs. G8	*	0.0121
	G4 vs. G7	*	0.0158
	G4 vs. G8	*	0.0216
	G6 vs. G7	**	0.0012
	G6 vs. G8	**	0.0016
8C- Females- *Prox1*	G1 vs. G2	**	0.0035
	G1 vs. G8	*	0.0315

	G2 vs. G3	***	0.0001
	G2 vs. G4	***	0.0004
	G2 vs. G5	***	0.0004
	G2 vs. G6	*	0.0285
	G2 vs. G7	***	0.0005
	G2 vs. G8	****	<0.0001
	G6 vs. G8	**	0.0039
8D-Females-*Vegfr2*	G3 vs. G8	*	0.0195
	G5 vs. G8	*	0.037
	G6 vs. G8	*	0.0224

Table S11: Comprehensive Summary of False Discovery Rate (FDR) Analysis Across All Experimental Cohorts.

Markers	Discovery?	P value	q value
**FIGURE 1-MALES**			
Fibrinogen	Yes	0.0001	0.0001
Adipsin	Yes	0.0001	0.0001
TNFα	Yes	0.0001	0.0001
RANTES/CCL5	Yes	0.0001	0.0001
MIG/CXCL9	Yes	0.0001	0.0001
**FIGURE 1-FEMALES**			
Fibrinogen	Yes	0.0007	0.0004
Adipsin	Yes	0.0173	0.0061
TNFα	Yes	0.0352	0.0092
RANTES/CCL5	Yes	0.0939	0.0197
MIG/CXCL9	Yes	0.0001	0.0001
**FIGURE 2-MALES**			
Hif1α	Yes	0.0002	0.0002
Redd1	Yes	0.0001	0.0001
HIF1α	Yes	0.0001	0.0001
GFAP	Yes	0.0001	0.0001
**FIGURE 2-FEMALES**			
Hif1α	Yes	0.0001	0.0001
Redd1	Yes	0.0318	0.0334
HIF1α	Yes	0.0001	0.0001
GFAP	Yes	0.0001	0.0001
**FIGURE 3-MALES**			
Vegfa	Yes	0.0012	0.0009
Vegfr2	Yes	0.0001	0.0002
Pdgfrβ	Yes	0.0013	0.0009
Aqp4	No	0.4275	0.1796
Rbp3	Yes	0.0621	0.0326
**FIGURE 3- FEMALES**			
Vegfa	Yes	0.0001	0.0002
Vegfr2	Yes	0.0001	0.0002
Pdgfrβ	Yes	0.0003	0.0004
Aqp4	Yes	0.0001	0.0002
Rbp3	Yes	0.0007	0.0007
**FIGURE 4- MALES**			
Foxo1	Yes	0.0008	0.0017
Pparα	Yes	0.0001	0.0003
Mcp-1	Yes	0.0031	0.0039
Nfκb	Yes	0.0001	0.0003
Il-1β	Yes	0.0377	0.0396

TNFα	Yes	0.0011	0.0017
**FIGURE 4- FEMALES**			
Foxo1	Yes	0.0006	0.0008
Pparα	Yes	0.0002	0.0003
Mcp-1	Yes	0.0001	0.0002
Nfκb	Yes	0.0001	0.0002
Il-1β	Yes	0.0001	0.0002
TNFα	Yes	0.0012	0.0013
**FIGURE 5- FEMALES**			
DVP-Total no of junctions	Yes	0.0001	0.0001
DVP-Vessel length	Yes	0.0001	0.0001
DVP-Mean Lacunarity	Yes	0.0002	0.0002
DVP-Vessel(%Area)	Yes	0.0001	0.0001
SVP-Total no of junctions	Yes	0.0001	0.0001
SVP-Vessel length	Yes	0.0001	0.0001
SVP-Mean Lacunarity	Yes	0.0001	0.0001
SVP-Vessel(%Area)	Yes	0.0005	0.0005
IBA(%Area)	Yes	0.0001	0.0001
GFAP(%Area)	Yes	0.0001	0.0001
**FIGURE 7- MALES**			
Heart Body weight ratio	Yes	0.0001	0.0001
MAP	Yes	0.0007	0.0007
LYVE (% Area)	Yes	0.0001	0.0001
Lyve1	Yes	0.0001	0.0001
PGC1α (% Area)	Yes	0.0001	0.0001
Pparα	Yes	0.0001	0.0001
**FIGURE 7- FEMALES**			
Heart Body weight ratio	Yes	0.0011	0.0012
MAP	Yes	0.0001	0.0001
LYVE (% Area)	Yes	0.0001	0.0001
Lyve1	Yes	0.0001	0.0001
PGC1α (% Area)	Yes	0.0001	0.0001
Pparα	Yes	0.0001	0.0001
**FIGURE 8- MALES**			
Vegfr3	Yes	0.0001	0.0001
Vegfc	Yes	0.0001	0.0001
Prox1	Yes	0.0003	0.0001
Vegfr2	Yes	0.0596	0.0156
**FIGURE 8- FEMALES**			
Vegfr3	Yes	0.0001	0.0002
Vegfc	Yes	0.0005	0.0007
Prox1	Yes	0.0001	0.0002
Vegfr2	Yes	0.0032	0.0034

Abbreviations

AQP4: Aquaporin-4

BRB: Blood-Retina Barrier

CD: Circular Dichroism

CPT1: Carnitine Palmitoyl Transferase 1

CUR: Curcumin

DR: Diabetic Retinopathy

FAS: Fatty Acid Synthase

FOXO1: Forkhead Box O1

GFAP: Glial Fibrillary Acidic Protein

HIF1α: Hypoxia-Inducible Factor 1 alpha

IL-1β: Interleukin-1 beta

INS: Insulin

IOP: Intraocular Pressure

IRS1: Insulin Receptor Substrate 1

LYVE1: Lymphatic vessel endothelial hyaluronan receptor 1

MAP: Mean Arterial Pressure

MCP-1: Monocyte chemoattractant protein-1

NFκB: Nuclear factor kappa-light-chain-enhancer of activated B cells

NLRP3: Nucleotide-Binding Oligomerization Domain (NOD)-like receptor family, pyrin domain-containing 3.

NVU: Neuro-Vascular Unit

PDGFRβ: Platelet-Derived Growth Factor Receptor beta

PGC1α: Peroxisome proliferator-activated receptor gamma coactivator 1-alpha

PLGA: Poly (lactic-co-glycolic acid)

PPARα: Peroxisome Proliferator-Activated Receptor alpha

PROX1: Prospero homeobox 1

RANTES: Regulated upon Activation, Normal T cell Expressed and Secreted

RBP3: Retinol Binding Protein 3

REDD1: Regulated in Development and DNA damage-response 1

STAT3: Signal Transducer and Activator of Transcription 3

STZ: Streptozotocin

s.c: subcutaneous

TNFα: Tumor Necrosis Factor alpha

TXNIP: Thioredoxin-Interacting Protein

VEGFA: Vascular Endothelial Growth Factor A

VEGFC: Vascular Endothelial Growth Factor C

VEGFR2: Vascular Endothelial Growth Factor Receptor 2

VEGFR3: Vascular Endothelial Growth Factor Receptor 3
